# Spent Coffee Ground Extracts: A Sustainable Source of Antioxidant and Immunomodulatory Bioactives for Managing Lifestyle-Related Chronic Diseases

**DOI:** 10.3390/ijms27114980

**Published:** 2026-05-30

**Authors:** Alifah Hasna, Belinda Anasthasya Tansy, Armansyah Maulana Harahap, Maulana Bagus Adi Cahyono, Edwin Hadinata, Raymond Rubianto Tjandrawinata, Fahrul Nurkolis, Lucia De Luca, Giulia Basile, Raffaele Romano, Antonello Santini

**Affiliations:** 1 Master of Basic Medical Science, Faculty of Medicine, Universitas Airlangga, Surabaya 60131, Indonesia; 2 School of Medicine, Faculty of Medicine, Universitas 17 Agustus 1945 (UNTAG) Surabaya, Surabaya 60118, Indonesia; 3 Department of Physical Education, Health and Recreation, Faculty of Sport, Universitas Negeri Medan, Medan 20221, Indonesia; 4 Medical Program, Faculty of Medicine, Universitas Airlangga, Surabaya 60132, Indonesia; 5 School of Medicine, Faculty of Medicine, Ciputra University of Surabaya, Surabaya 60219, Indonesia; 6 School of Bioscience, Innovation and Technology, Atma Jaya Catholic University of Indonesia, Jakarta 12930, Indonesia; 7 Medical Research Center of Indonesia, Surabaya 60281, Indonesia; 8 Institute for Research and Community Service, State Islamic University of Sunan Kalijaga (UIN Sunan Kalijaga), Yogyakarta 55281, Indonesia; 9 Department of Agricultural Sciences, University of Napoli Federico II, Piazza Carlo di Borbone 1, 80055 Portici, Italy; 10 Department of Pharmacy, University of Napoli Federico II, Via Domenico Montesano, 49, 80131 Napoli, Italy

**Keywords:** spent coffee grounds, chronic inflammation, polyphenols, ion channel modulation, G-protein-coupled receptors, immunometabolic regulation, oxidative stress, nutraceuticals

## Abstract

This review aims to comprehensively examine spent coffee grounds (SCGs) as a sustainable source of antioxidant and immunomodulatory bioactives, with a specific focus on their capacity to modulate membrane-level signaling through ion channels and G-protein-coupled receptors (GPCRs) in the context of lifestyle-related chronic diseases. SCGs, the major solid by-product of coffee brewing, represent an underutilized yet highly abundant source of bioactive compounds, including chlorogenic acids, phenolic acids, melanoidins, diterpenes, and residual alkaloids. Lifestyle-related chronic diseases, including type 2 diabetes, obesity, cardiovascular disease, and chronic inflammatory disorders, are increasingly recognized as immunometabolic conditions driven by persistent low-grade inflammation, redox imbalance, and dysregulated membrane signaling. This review synthesizes current evidence demonstrating that bioactives contained in SCG extracts exert antioxidant and immunomodulatory effects that extend beyond radical scavenging. Crucially, these compounds also act as modulators of membrane-level signaling, representing a mechanistic perspective that has not been previously integrated for SCGs in the context of chronic disease. The different extraction methodologies and the obtained results are evaluated with the aim to identify the most effective experimental approach and extraction conditions. The paper also discusses how SCG compounds regulate redox-sensitive ion channels (including calcium channels, TRP channels, and potassium channels), and key GPCR pathways (such as GPR120, GPR43, and adenosine receptors), thereby influencing immune cell activation, cytokine production, insulin signaling, and metabolic inflammation. Particular attention is given to the role of microbial fermentation and enzymatic processing in enhancing SCG bioavailability, generating postbiotic metabolites that further engage GPCR–ion channel crosstalk. By integrating extraction approaches, antioxidant chemistry, immunology, membrane signaling, and nutritional metabolism, this review positions SCG as a sustainable functional ingredient capable of restoring immune tolerance and metabolic homeostasis. These insights support the valorization of SCGs within the circular economy framework and highlight their potential application in next-generation immunonutrition strategies for chronic disease prevention and management.

## 1. Introduction

Lifestyle-related chronic diseases such as type 2 diabetes (T2D), obesity, cardiovascular disease (CVD), and other chronic inflammatory disorders are now understood as complex immunometabolic disorders. These conditions are characterized not only by metabolic dysfunction but also by persistent immune dysregulation, oxidative stress, and aberrant membrane signaling. In T2D and obesity, for example, chronic low-grade inflammation (“metaflammation”) driven by immune cells and inflammatory cytokines plays a pivotal role in pathogenesis [1,2]. Metaflammation represents a state of chronic, subthreshold activation of innate immune pathways in metabolic tissues, triggered by nutrient excess rather than infection, and is now recognized as a central driver of insulin resistance and disease progression [2]. Inflammatory signaling, particularly TNF-α, directly impairs insulin receptor function, establishing a molecular link between immunity and metabolism that has since become foundational to the field. Individuals with T2D exhibit elevated levels of pro-inflammatory cytokines (e.g., IFN-γ, TNF-α, IL-6, IL-17) that contribute to insulin resistance and vascular complications [1]. Concurrently, these diseases feature redox imbalance, with excessive reactive oxygen species (ROS) sustaining inflammation (inflammaging) and damaging tissues. Indeed, in metabolic syndrome and neuroinflammation, oxidative and even reductive stress can both disrupt immune homeostasis, fueling chronic inflammation [3,4,5]. Importantly, this redox imbalance does not act in isolation; ROS can directly oxidize and dysregulate membrane-bound proteins, including ion channels and receptor complexes, thereby corrupting the electrochemical signals that govern immune cell activation and metabolic responses. This oxidative disruption of membrane architecture provides a mechanistic bridge between systemic redox imbalance and the broader dysfunction of cell-surface signaling observed in chronic disease. Another emerging layer is membrane signaling dysfunction: dysregulated activity of cell-surface receptors and ion channels has been implicated in these disorders. Recent research highlights that disturbances in ion homeostasis and channel function are closely linked to the development of hypertension, diabetes, obesity, and atherosclerosis [6]. In other words, defects at the membrane level (such as aberrant G-protein-coupled receptor (GPCR) and ion channel signaling) interact with metabolic and immune pathways in chronic disease. This presents a gap in nutraceutical research; while most studies focus on antioxidants as enzyme inhibitors or gene regulators, fewer have addressed how bioactive compounds modulate membrane-level signaling (ion fluxes, GPCR activation) that underlies immune–metabolic crosstalk.

The present review is motivated by a critical gap in the existing literature: while the health benefits of brewed coffee have been extensively studied, the bioactive potential of SCG and specifically its capacity to modulate membrane-level immunometabolic signaling has received comparatively little systematic attention. Most prior reviews of spent coffee grounds (SCGs) have focused on compositional characterization or antioxidant capacity, without integrating the emerging evidence on ion channel and GPCR modulation. This review addresses that gap by adopting a membrane-centric framework to evaluate SCG bioactivity.

The objectives of this review are therefore fourfold, (1) to evaluate and compare extraction methodologies for SCG bioactives, identifying the most effective approaches for recovering functionally active compounds; (2) to explore antioxidant mechanisms of SCG constituents beyond direct radical scavenging, with emphasis on redox modulation at the membrane level; (3) to characterize the role of specific ion channels (calcium channels, TRP channels, and potassium channels) and GPCRs (GPR120, GPR43, and adenosine receptors) as molecular targets of SCG bioactives in immunometabolic regulation; and (4) to discuss the translational potential of SCG within precision nutrition and circular economy frameworks, including safety, standardization, and regulatory considerations.

By integrating evidence from extraction science, antioxidant biochemistry, immunology, and membrane biophysics, this review positions SCG not merely as a coffee by-product, but as a multi-target functional ingredient capable of addressing the convergent pathological mechanisms (oxidative stress, metaflammation, and membrane signaling dysfunction) that underlie lifestyle-related chronic diseases. This integrated perspective has not been previously synthesized for SCGs and represents the primary novelty of the present work.

### 1.1. Lifestyle-Related Chronic Diseases as Immunometabolic Disorders

Mounting evidence indicates that chronic diseases of lifestyle are driven by integrated immune and metabolic dysfunction. Obesity and T2D, for instance, are accompanied by an accumulation of pro-inflammatory immune cells (e.g., M1 macrophages, Th1/Th17 cells) in adipose and other tissues, creating a state of systemic inflammation [1,2]. This “low-grade” inflammation is subclinical yet persistent, disrupting insulin signaling and contributing to tissue damage. Adipose tissue in obesity produces excess TNF-α, IL-6, and other cytokines, which interfere with insulin receptor signaling and glucose uptake [1]. The concept of metaflammation has emerged to describe how nutrient excess and metabolic stress trigger immune activation. Metaflammation is characterized by chronic activation of innate immune pathways in metabolic tissues, without overt infection [2,7,8]. This immune activation links to redox imbalances: overnutrition and hyperglycemia elevate ROS via mitochondrial overload and NADPH oxidase, activating redox-sensitive inflammatory pathways like NF-κB. Notably, oxidative stress and inflammation reinforce each other in a vicious cycle [3]. At the same time, recent findings highlight that ion channels are important immunometabolic regulators. Calcium (Ca^2+^) and potassium (K^+^) channels on immune cells influence their activation and cytokine release, as discussed later, and metabolic diseases show altered ion channel expression or activity [6,9,10]. For example, disrupted Ca^2+^ homeostasis has been linked to insulin resistance and inflammation in diabetes [3,11]. Thus, chronic lifestyle diseases can be seen as a convergence of metabolic derangements, chronic inflammation, oxidative stress, and membrane signaling dysfunction at the level of receptors and channels. Nutritional interventions that address only antioxidant capacity or enzyme inhibition may therefore overlook critical therapeutic targets at the cell membrane, a limitation that motivates the membrane-centric approach adopted in this review.

### 1.2. Coffee Consumption vs. Spent Coffee Grounds: The Missing Half

**Figure 1:**
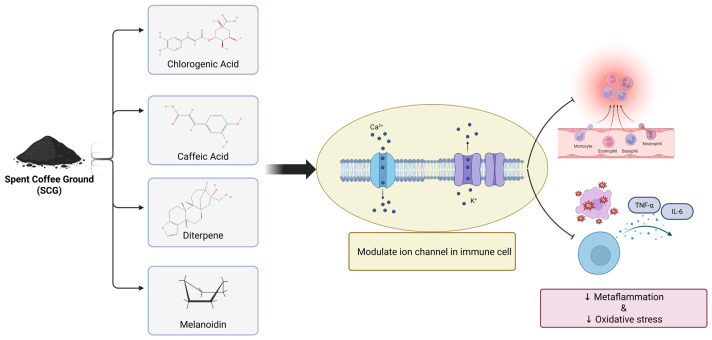
Conceptual framework illustrating spent coffee grounds (SCGs) as a membrane-centric immunometabolic modulator. Bioactive compounds retained in SCGs, including polyphenols, melanoidins, diterpenes, and fermentation-derived short-chain fatty acids (SCFAs), target cell membrane signaling by modulating ion channels and G-protein-coupled receptors (GPCRs). Through coordinated regulation of Ca^2+^ and K^+^ fluxes and anti-inflammatory GPCR pathways, SCG bioactives restore redox balance, dampen metaflammation, and promote immune tolerance, thereby improving metabolic and inflammatory outcomes in lifestyle-related chronic diseases. Created in Biorender. Fahrul Nurkolis. (2026). https://biorender.com (accessed on 18 April 2026).

Coffee is a widely consumed beverage known for its rich phytochemistry and potential health benefits (e.g., lower risk of T2D and CVD) [12]. These benefits are often attributed to bioactive compounds like chlorogenic acids (CGAs) and caffeine present in the brew (Figure 1). However, an often-neglected fact is that brewing coffee leaves behind a large mass of residue known as spent coffee grounds (SCGs). Remarkably, nearly half or more of the original coffee biomass remains as SCG after extraction of the beverage; some estimates indicate SCGs can constitute up to ~80% of the roasted coffee bean’s mass [12,13,14]. Globally, about 6–8 million tons of SCGs are generated annually, and this residue is typically treated as waste, sent to landfills or incinerated with environmental consequences (greenhouse gas emissions, pollution). Yet, SCGs should not be viewed merely as waste. Brewing removes only water-soluble components; the spent grounds retain a wealth of bioactive constituents that make them an underexplored functional biomass. SCGs are rich in diverse classes of compounds including phenolic acids, diterpenoid lipids, melanoidins, proteins, polysaccharides, and minor alkaloids [12,15,16]. Key nutraceutical compounds such as CGAs, caffeic and ferulic acid (phenolics), and coffee-specific diterpenes (cafestol and kahweol) are present in significant amounts in SCGs [12,17]. Additionally, roasting-generated melanoidin’s brown polymeric compounds with antioxidant activity remain in the spent grounds. In essence, up to 50% or more of coffee’s bioactive potential may lie in the leftover grounds. This “missing half” of coffee’s health benefits has been underutilized, and its potential to modulate immune–metabolic pathways through membrane signaling mechanisms remains largely unexplored. Unlocking SCG’s functional compounds could yield sustainable nutraceutical ingredients, aligning with circular economy principles (converting “waste” to value) and providing novel interventions for chronic disease management.

Therefore, this review aims to comprehensively examine spent coffee grounds as a sustainable source of antioxidant and immunomodulatory bioactives, with a specific focus on their ability to modulate ion channels and GPCR-mediated membrane signaling. The novelty of this work lies in reframing SCG bioactivity beyond conventional antioxidant paradigms, highlighting coordinated ion channel–GPCR crosstalk as a unifying mechanism linking redox regulation, immune tolerance, and metabolic homeostasis in lifestyle-related chronic diseases.

## 2. Bioactive Extraction in Spent Coffee Grounds

Efficient extraction of bioactive compounds from spent coffee grounds (SCGs) is a critical step that determines both the yield and functional quality of the recovered molecules. Given the complex and heterogeneous matrix of SCGs, where phenolics are often bound to lignocellulosic structures and melanoidins, the choice of extraction technique significantly influences the recovery of antioxidant and immunomodulatory compounds. Conventional solvent-based extraction (e.g., maceration or Soxhlet using water, ethanol, or hydroalcoholic mixtures) has been widely employed due to its simplicity and scalability. However, these approaches often show limited efficiency in releasing bound phenolics and may require long extraction times and high solvent consumption.

To overcome these limitations, advanced extraction techniques have been increasingly explored. Ultrasound-assisted extraction (UAE) enhances mass transfer through acoustic cavitation, disrupting plant cell walls and improving the release of phenolic compounds, including CGAs and caffeic acid derivatives. Similarly, microwave-assisted extraction (MAE) accelerates solvent penetration and promotes rapid heating, resulting in higher extraction yields within shorter processing times. Both UAE and MAE have demonstrated superior efficiency compared to conventional methods, particularly in maximizing total phenolic content (TPC) and antioxidant capacity (e.g., DPPH and FRAP assays), while reducing energy and solvent usage.

Supercritical fluid extraction (SFE), typically using CO_2_ with ethanol as a co-solvent, represents another promising green technology for selectively extracting lipophilic bioactives such as diterpenes (cafestol and kahweol). SFE offers advantages in terms of solvent-free extracts and tunable selectivity; however, its effectiveness for polar phenolics is limited unless modifiers are employed. Enzyme-assisted extraction (EAE) has emerged as a particularly effective strategy for SCGs, given the high proportion of bound polyphenols. Enzymes such as cellulases, hemicellulases, and mannanases facilitate the breakdown of polysaccharide–polyphenol complexes, significantly enhancing phenolic release. For example, enzymatic hydrolysis has been reported to increase phenolic yield by several folds compared to non-treated SCGs, highlighting its potential as a key pre-treatment step.

In addition, biotechnological approaches such as microbial fermentation further improve extraction efficiency and bioavailability. Solid-state fermentation using fungi or bacteria not only enhances the liberation of phenolic compounds but also generates secondary metabolites with improved antioxidant and immunomodulatory properties. These processes can lead to increased levels of hydroxycinnamic acids and bioactive metabolites that are otherwise inaccessible in raw SCGs.

Current evidence suggests that no single extraction technique is universally optimal; rather, integrated or sequential approaches (e.g., enzymatic pre-treatment followed by UAE or MAE) appear to provide the most efficient recovery of SCG bioactives. Conventional solvent extraction remains useful for baseline recovery, whereas advanced and hybrid techniques offer superior performance in terms of yield, selectivity, sustainability, and functional bioactivity. Therefore, the selection of extraction strategy should be tailored to the target compound class (phenolics vs. lipophilic compounds) and intended application (nutraceutical, functional food, or pharmaceutical use), with emerging green extraction technologies representing the most promising direction for future SCG valorization.

### 2.1. Major Antioxidant Constituents in SCGs

Polyphenols: Spent coffee grounds contain a substantial fraction of coffee’s original polyphenols, both in free form and bound to insoluble structures. The most abundant are the CGAs, a family of caffeoyl-quinic acid conjugates (including 5-CQA, 3-CQA, etc.). CGAs and their derivatives (caffeic, ferulic, and p-coumaric acids) are potent antioxidants and contribute to coffee’s health effects. In SCGs, a portion of CGAs remains unextracted; moreover, some CGA fragments become incorporated into high-molecular-weight melanoidins during roasting [12,18,19]. Caffeic acid and ferulic acid, released from CGAs or present in bound form, have well-documented radical scavenging and anti-inflammatory activities. SCG extracts have shown significant total phenolic content and antioxidant capacity, indicating these polyphenols are still present after brewing. Notably, studies demonstrate that enzymatic treatments can liberate phenolics from SCGs; e.g., β-mannanase hydrolysis increased phenolic yield ~6-fold (from ~46 to 292 mg GAE/g) by breaking polysaccharide–polyphenol linkages [12,20]. This highlights that a substantial polyphenol reservoir in SCGs is bound or entrapped, and specialized processing can unleash its antioxidant potential.

Melanoidins: These are complex Maillard reaction products formed during coffee roasting. Melanoidins account for a significant portion of SCG solids (estimated ~13–25% of SCG dry mass). Chemically, coffee melanoidins are heterogeneous polymers composed of browned polysaccharides, proteins, and incorporated phenolics. Far from being inert, melanoidins have multiple bioactivities. They exhibit strong antioxidant capacity in vitro, acting as radical scavengers and metal chelators [21,22,23]. Importantly, melanoidins can behave as redox buffers, mitigating oxidative stress by sequestering reactive species. Unlike simple antioxidants, melanoidins have a high MW and resist digestion; hence, they reach the colon intact, functioning as dietary fiber. In the gut, melanoidins are fermented by microbiota, releasing phenolic fragments and short-chain fatty acids (SCFAs) that have systemic antioxidant and anti-inflammatory effects. Coffee melanoidins also have immunomodulatory interactions: for example, they can inhibit bacterial adhesion and modulate gut microbiota (promoting beneficial bifidobacteria). Melanoidins from SCGs thus act as immune-interacting polymers as they not only neutralize ROS but also influence immune signaling (e.g., by binding endotoxins or regulating colonic enzymes) [21,22,24]. These properties underscore that melanoidins are not passive antioxidants but actively participate in maintaining redox and immune homeostasis.

Alkaloids and Minor Lipophilic Compounds: Spent grounds retain some alkaloids, chiefly caffeine. While most caffeine is extracted into the brew, SCGs can contain anywhere from ~0.1% up to 0.4% caffeine by dry weight depending on brewing conditions. Caffeine is a notable biologically active compound; it is a known adenosine receptor antagonist and CNS stimulant, and is often included as an analgesic adjuvant due to its vasoactive and neuromodulatory effects [12,25,26]. In an immunological context, caffeine’s blockade of adenosine A2A receptors on immune cells can modulate cytokine production and inflammatory cell migration (with complex outcomes discussed later). SCGs also contain diterpenes such as cafestol and kahweol, lipid-soluble molecules originally in coffee oil. Although brewing with paper filters removes most diterpenes, SCGs (especially from unfiltered methods or espresso) will still harbor these compounds in their oils [17,27]. Cafestol and kahweol are noteworthy because they have been found to exhibit pharmacological activities including anti-inflammatory and antioxidant effects despite being infamously associated with raising cholesterol. Research indicates these coffee diterpenes suppress NF-κB activation and downregulate pro-inflammatory cytokines like IL-8 and TNF-α in various cells [28,29]. Thus, SCG’s minor lipophilic fraction, though small, contains bioactives that can influence inflammatory pathways.

In summary, SCGs provide a complementary profile of coffee’s bioactives: rich phenolic acids (both free and bound), melanoidin–polyphenol complexes acting as macromolecular antioxidants, and alkaloid and diterpene compounds. Table 1 outlines key SCG constituents and their known antioxidant/immunomodulatory effects. These constituents work in concert, positioning SCGs as a source of multi-functional nutraceutical compounds capable of modulating redox status and immune signaling at different biological levels.

### 2.2. Bioavailability and Fermentation-Enhanced Activity

A crucial consideration for SCG bioactives is their bioavailability. Many phenolics in SCGs are insoluble or bound within fibers, potentially limiting their absorption in native form. However, biological processing (either prior to consumption or via gut microbiota) can enhance the release and efficacy of these compounds. One strategy is enzymatic hydrolysis or tailored extraction. As noted, enzymatic pre-treatment of SCGs (with carbohydrases like mannanases or glucosidases) can free phenolic acids from the matrix, dramatically increasing the extractable antioxidant content [12,32,34]. This approach effectively “pre-digests” the fiber–phenol complexes, yielding smaller phenolics that are more bioaccessible. Another strategy harnesses microbial fermentation. For instance, solid-state fermentation of SCGs using fungi (e.g., *Trichoderma* or *Rhizopus* species) has been shown to increase phenolic yields and antioxidant activity. Fermenting microbes produce their own enzymes that break down polysaccharides and liberate polyphenols. One study found that fermenting SCGs with certain fungi significantly elevated levels of hydroxycinnamic acids (like caffeic and ferulic acid) compared to unfermented SCGs. These released compounds greatly enhanced the DPPH and FRAP (antioxidant) activities of the extracts [35,36,37]. Thus, bioprocessing SCGs can yield “fermentation-enhanced” nutraceutical ingredients, with higher antioxidant potency and potentially novel metabolites.

In the gastrointestinal tract, the gut microbiota performs a natural fermentation of SCG dietary fiber and melanoidins. SCG components that resist upper gut digestion (insoluble fiber, melanoidins) reach the colon, where bacteria metabolize them into metabolites such as SCFAs (e.g., acetate, propionate, butyrate) and various phenolic catabolites (e.g., dihydrocaffeic acid from CGAs). These microbial metabolites significantly contribute to SCG’s bioactivity. SCFAs in particular have systemic effects: they act as signaling molecules via free fatty acid receptors (like GPR43/GPR41) on immune and endocrine cells. SCG fermentation yields SCFAs that can mimic some benefits of dietary fiber from other sources including improving colonic regulatory T cell responses, enhancing gut barrier integrity, and modulating metabolism (like increasing GLP-1 secretion which improves insulin sensitivity). Indeed, the degradation of SCG melanoidins in the colon produces acetate and butyrate associated with anti-inflammatory properties [21,38,39]. These SCFAs engage a gut–immune–endocrine axis; e.g., activation of GPR43 on neutrophils and adipocytes reduces inflammatory responses and stimulates adiponectin secretion, respectively. Additionally, microbial breakdown of SCG polyphenols generates smaller phenolic acids (e.g., feruloyl and caffeoyl derivatives) that can be absorbed and exert systemic antioxidant effects (such as crossing the blood–brain barrier and reducing neuroinflammation). In essence, the gut microbiome acts as a bioreactor converting SCG’s complex macromolecules into more bioactive and bioavailable compounds [40,41].

In summary, while raw SCGs contain abundant bioactives, their full potential is unlocked through enzymatic processing or microbial fermentation. These processes not only increase the quantity of extractable antioxidants but also generate metabolites that directly engage immune and metabolic signaling pathways. SCG thus can be transformed from an insoluble residue into a source of readily bioactive compounds that target key nodes in chronic disease (redox balance, GPCRs, ion channels, etc.), as explored in subsequent sections.

## 3. Antioxidant Mechanisms Beyond Radical Scavenging

Traditional assessments of antioxidants often focus on direct radical scavenging capacity (e.g., neutralizing DPPH or hydroxyl radicals). However, emerging insight suggests that effective antioxidative therapy in chronic diseases involves modulating redox signaling at the cellular and membrane level, not merely quenching free radicals in a test tube [42]. The bioactives in spent coffee grounds likely exert much of their benefit by regulating redox-sensitive signaling pathways and ion fluxes in cells. In other words, they function as redox modulators that stabilize cellular homeostasis under stress, rather than acting as simple chemical antioxidants.

### Redox Modulation at the Membrane Level

One novel framing is that antioxidants can serve as ion flux stabilizers. Oxidative stress and Ca^2+^ signaling are intimately linked: excessive ROS can dysregulate ion channels, and abnormal Ca^2+^ influx can, in turn, trigger ROS production (for instance, via mitochondrial dysfunction). SCG bioactives target these processes to prevent a self-perpetuating cycle of oxidative damage and inflammation. For example, SCG polyphenols (like CGAs) have been shown to attenuate Ca^2+^ influx into cells during stress conditions. High oxidative stress typically causes Ca^2+^ overload; ROS can activate certain Ca^2+^ channels or damage membranes leading to Ca^2+^ leak. This is seen in ischemic tissues where dysfunctional mitochondria release ROS and fail to buffer Ca^2+^, resulting in cytosolic Ca^2+^ spikes that activate destructive enzymes [43,44]. By scavenging ROS and upregulating endogenous antioxidants, CGAs and related compounds indirectly keep Ca^2+^ channels (like CRAC or TRP channels) from aberrant activation. Additionally, some polyphenols may directly inhibit specific Ca^2+^ channels; there is evidence that CGAs can suppress voltage-dependent Ca^2+^ currents in vascular cells, contributing to vasodilatory and antioxidant effects [30,45,46]. Thus, rather than purely neutralizing radicals, these compounds prevent ROS-induced Ca^2+^ dysregulation, protecting cells from Ca^2+^-triggered inflammatory cascades.

Moreover, SCG bioactives modulate the feedback loops of redox signaling. For instance, mitochondrial ROS-Ca^2+^ coupling is a crucial node: moderate increases in mitochondrial Ca^2+^ stimulate metabolism, but excessive Ca^2+^ causes overproduction of ROS and cell death. Antioxidants from SCGs help maintain this balance. By limiting mitochondrial ROS, they prevent the opening of ROS-sensitive Ca^2+^ channels (like the mitochondrial permeability transition pore or TRPM2 in the plasma membrane) [47,48]. This keeps mitochondrial membrane potential stable and avoids the catastrophic Ca^2+^ release that would otherwise occur under oxidative stress. Studies on immune cells show that TRPM2, a calcium-permeable channel activated by ROS, plays a dual role in inflammation; it can promote cytokine release when overactivated [48,49]. SCG compounds, by reducing ROS levels, effectively keep TRPM2 activity in check, thereby dampening downstream inflammatory signals such as NLRP3 inflammasome activation [50].

Another membrane-level target is the family of NADPH oxidase (NOX) enzymes, which are major sources of ROS in immune cells and endothelium. NOX enzymes reside in cell membranes and pump out superoxide in response to stimuli. Interestingly, some polyphenols act as indirect NOX inhibitors. CGAs, for example, have been shown to suppress NOX activity in vascular cells, reducing oxidative burst [30,31]. SCG-derived CGAs and melanoidin-bound phenolics likely contribute to NOX attenuation, thereby preventing the initiation of redox-driven inflammatory pathways. By curbing NOX-derived ROS, they not only reduce oxidative damage but also prevent ROS from activating redox-sensitive ion channels and transcription factors (like NF-κB) [51,52]. This suggests a mechanism where SCG antioxidants interrupt the feed-forward loop, metabolic stress → NOX activation → ROS → Ca^2+^ influx and NF-κB activation → inflammatory gene expression. Instead, with NOX kept at bay, cells maintain redox homeostasis even under metabolic challenge.

Furthermore, SCG compounds may influence membrane lipid organization and receptor function in ways that affect redox signaling. The coffee diterpenes, despite their lipid nature, can insert into membranes and modulate signaling complexes. For instance, cafestol has been noted to activate certain Nrf2-mediated antioxidant responses and might integrate into lipid rafts, altering receptor localization. Such actions can enhance the cell’s antioxidant defenses on the signaling level, not just by chemical quenching of ROS [29,53].

**Figure 2:**
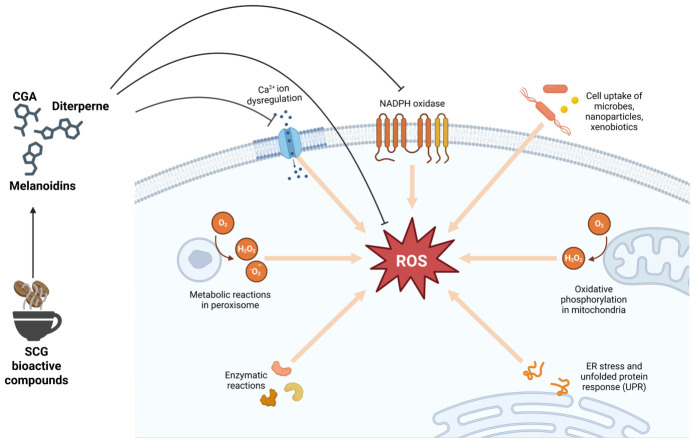
Antioxidant mechanisms of spent coffee ground bioactives beyond direct radical scavenging. SCG polyphenols and melanoidin-bound antioxidants modulate redox signaling at the membrane and mitochondrial levels by suppressing NADPH oxidase activity, stabilizing Ca^2+^ homeostasis, and preventing ROS-driven inflammatory amplification loops, thereby preserving cellular homeostasis under metabolic stress. Created in Biorender. Fahrul Nurkolis. (2026). https://biorender.com (accessed on 18 April 2026).

In summary, the antioxidant effect of spent coffee ground bioactives extends beyond direct radical scavenging (Figure 2). They help regulate ion channel gating and receptor signaling under oxidative conditions. By stabilizing Ca^2+^ homeostasis, preventing uncontrolled ion fluxes, and inhibiting ROS-generating membrane enzymes like NOX, SCG compounds protect cells from oxidative injury and interrupt pro-inflammatory signal transduction. This nuanced redox modulation is particularly relevant to chronic diseases, where continuous oxidative and inflammatory signals disturb cellular equilibrium. SCG bioactives act to restore this equilibrium at the membrane and cytosolic signaling level. In the next sections, we explore how these effects translate to specific ion channel and GPCR targets involved in immunometabolic regulation.

## 4. Ion Channels as Immunometabolic Targets

**Figure 3:**
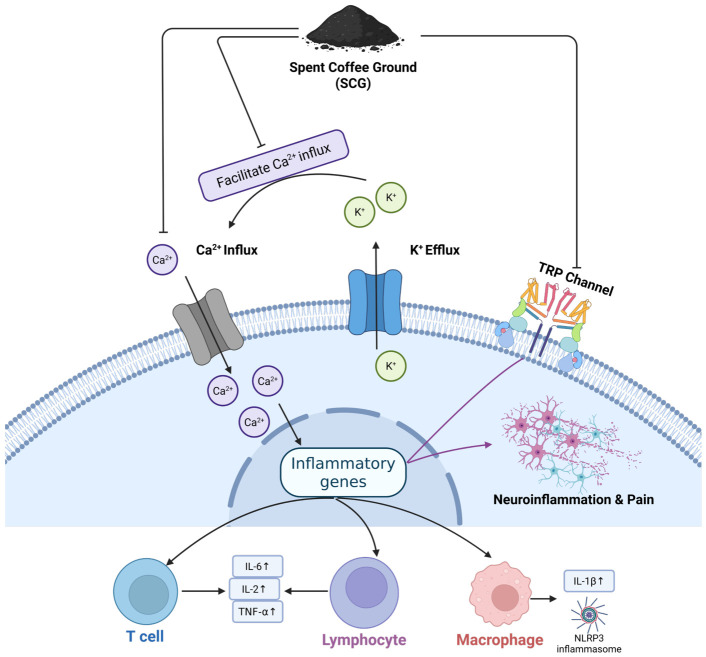
Ion channel-mediated immunometabolic regulation by spent coffee ground bioactives. SCG-derived compounds modulate calcium and potassium channels, including CRAC, TRP channels (TRPV1, TRPA1, TRPM2), Kv1.3, and KATP channels, leading to controlled immune cell activation, reduced cytokine release, protection of pancreatic β-cells, and attenuation of neurogenic and metabolic inflammation. Created in Biorender. Fahrul Nurkolis. (2026). https://biorender.com (accessed on 18 April 2026).

Ion channels are emerging as key therapeutic targets in metabolic and inflammatory diseases, linking electrical and ionic signals with immune cell function and metabolism [14,54]. Many immune and metabolic cells rely on tightly controlled ion fluxes (especially Ca^2+^ and K^+^) for their activation and signaling (Figure 3). Dysregulation of these channels can lead to aberrant inflammatory responses or metabolic imbalances. Nutraceutical compounds from SCGs are poised to influence several of these channels, thereby exerting immunomodulatory effects. This section highlights major ion Ca^2+^ channels, TRP channels, and K^+^ channels and how SCG bioactives can modulate them to foster a healthier immune–metabolic state.

### 4.1. Calcium Channels and Immune Activation

Calcium influx is a central trigger for immune cell activation. Upon antigenic or inflammatory stimulation, a rise in cytosolic Ca^2+^ in lymphocytes and macrophages is necessary for the production of cytokines (like IL-2, IL-6, TNF-α) and other immune functions. This Ca^2+^ typically enters through calcium release-activated calcium (CRAC) channels (comprising ORAI1 proteins) and other Ca^2+^ channels following ER calcium store depletion or receptor signaling. T cells, for instance, absolutely require Ca^2+^ influx through CRAC channels to activate the transcription factor NFAT and express IL-2 and other cytokines; CRAC channel deficiency causes an inability to produce a broad spectrum of cytokines (IL-2, IL-4, IFN-γ, TNF-α, etc.) [55,56]. Similarly, macrophages utilize Ca^2+^ signaling for the triggering of inflammasomes and secretion of IL-1β. Thus, overactive Ca^2+^ signaling can lead to a hyper-inflammatory phenotype, while controlled attenuation of Ca^2+^ entry can yield an anti-inflammatory effect.

Polyphenols in spent coffee grounds appear to mildly attenuate Ca^2+^ overactivation in immune cells, thereby promoting an anti-inflammatory phenotype. While they likely do not block calcium channels outright (as immunosuppressive drugs like cyclosporine or CRAC inhibitors would), SCG compounds reduce the upstream triggers of excessive Ca^2+^ influx. For example, as discussed, CGAs suppress ROS and prevent the aberrant activation of Ca^2+^ channels like CRAC and TRPM2 that are stimulated under oxidative stress. By keeping oxidative tone low, CGA indirectly keeps Ca^2+^ influx to the necessary minimum (enough for normal function but not so much as to trigger massive cytokine release). Additionally, some SCG compounds may interact with signaling pathways that lead to Ca^2+^ influx. Caffeic acid, for instance, inhibits PKC and MAPK pathways in monocytes; these pathways normally potentiate Ca^2+^ entry and NF-κB activation. By dampening these signals, caffeic acid effectively reduces the magnitude of Ca^2+^-dependent cytokine gene transcription [57,58,59].

One notable target is the store-operated Ca^2+^ entry (SOCE) mechanism via CRAC channels (*ORAI1*/*STIM1*). T cells with overstimulated SOCE produce excess inflammatory cytokines, whereas partial SOCE inhibition skews them to a regulatory response [55,60]. SCG-derived molecules, by reducing SOCE-driving signals (like IP3 generation or oxidative modifications of *STIM1*), can temper T cell activation. The outcome is a shift from a highly inflammatory state to a more controlled state. Indeed, studies of polyphenol-rich diets show decreased T cell proliferation and a tilt towards anti-inflammatory cytokines effects analogous-to-mild CRAC channel modulation. It is plausible that SCG polyphenols contribute to such effects. For example, ferulic acid has been reported to inhibit T cell proliferation in vitro, consistent with limiting Ca^2+^-dependent IL-2 production [41,61].

Another set of Ca^2+^ channels relevant to immunometabolism are the voltage-gated Ca^2+^ channels (VGCCs) present in some immune cells and pancreatic β-cells. VGCCs in T cells are less well-defined than CRAC, but there is evidence for their involvement in certain T cell functions [55]. Nutrients like coffee compounds could influence these as well. If SCG diterpenes or polyphenols hyperpolarize the membrane (via K^+^ channel activation, see Kv1.3 below), they indirectly reduce VGCC opening (since these channels need depolarization to open) [45,54]. This would decrease Ca^2+^ influx and subsequent activation signals. Thus, SCG bioactives might indirectly modulate Ca^2+^ channel activity by altering membrane potential or second messenger levels.

The net result is that SCG compounds can help prevent Ca^2+^ overactivation in immune cells, leading to lower production of pro-inflammatory cytokines and a more quiescent immune state. In macrophages, this manifests as reduced release of IL-1β and IL-6 (since Ca^2+^ is needed for inflammasome and secretion processes). In T cells, it means somewhat lower IL-2 and IFN-γ, favoring regulatory T cell responses. By attenuating the Ca^2+^-NFAT/NF-κB pathway, SCG bioactives essentially put a brake on the immune activation that drives chronic inflammation. This is a nuanced immune modulation not an outright immunosuppression, but a rebalancing toward anti-inflammatory signaling.

### 4.2. TRP Channels: Redox-Sensitive Gates in Pain and Metabolism

Transient Receptor Potential (TRP) channels are a family of ion channels that act as sensors of the cellular environment, including temperature, pain stimuli, and oxidative stress. Several TRP channels are redox-sensitive and have been implicated in inflammation, pain perception, and metabolic regulation. Among these, TRPV1, TRPA1, and TRPM2 are particularly relevant to lifestyle-related diseases:

1. TRPV1 (Transient Receptor Potential Vanilloid 1): Famously known as the capsaicin receptor, it is a polymodal cation channel responsive to heat, acidity, and certain lipids. TRPV1 is expressed not only on sensory neurons (where it mediates pain and neurogenic inflammation) but also on some immune and metabolic cells (e.g., pancreatic islet cells, adipose nerves) [62]. Intriguingly, TRPV1 plays a complex role in metabolism studies in mice, showing that TRPV1 influences insulin secretion and energy expenditure. Genetic deletion of TRPV1 can exacerbate diet-induced obesity and insulin resistance, suggesting TRPV1 activity has protective metabolic effects (possibly through enhanced sympathetic burn-off of calories and improved insulin signaling) [63]. On the other hand, chronic overstimulation of TRPV1 (as in chronic pain) leads to neuroinflammation and stress (e.g., in type 1 diabetes, TRPV1 overactivity contributes to neuropathy and inflammation) [62]. Therefore, a balanced modulation (“Goldilocks effect”) of TRPV1 is desirable enough to reap metabolic benefits but not so much as to cause chronic pain/inflammation.
  i. Coffee bioactives and TRPV1: While SCGs do not contain capsaicin, certain compounds in coffee may indirectly affect TRPV1 pathways. Caffeine, for example, raises catecholamine levels which activate TRPV1-expressing brown adipose tissue, potentially aiding thermogenesis (similar to how capsinoids work) [63]. CGAs has been reported to upregulate adiponectin and reduce obesity-induced inflammation, effects that might intersect with TRPV1 signaling networks (since TRPV1 in sensory fibers can modulate adipose tissue inflammation via neuropeptides). Furthermore, the SCFA produced from SCG fiber fermentation could activate GPR41/43 on sensory neurons, which crosstalk with TRPV1 sensitization or desensitization. The desensitization of TRPV1 rendering it less responsive after initial activation is a mechanism exploited by capsaicin to relieve pain. It is conceivable that some SCG compounds might promote TRPV1 desensitization (for instance, by mild activation of TRPV1 or by activating PKA/CAMKII pathways that phosphorylate and desensitize the channel). If so, this would reduce chronic pain signaling and neurogenic inflammation (beneficial in conditions like diabetic neuropathy). Indeed, epidemiological data link coffee intake with lower risk of chronic pain conditions and improved neuropathic pain outcomes, hinting at TRPV1 involvement.
  ii. Overall, SCG bioactives likely “tone” TRPV1 activity supporting its metabolic benefits (enhanced thermogenesis, insulin release) while preventing overactivation. The outcome could be reduced inflammatory pain (through TRPV1 desensitization in nociceptors) and improved glucose handling (TRPV1-mediated insulinotropic effect in β-cells) [63,64]. This TRPV1 modulation by coffee compounds is a novel angle on how diet influences pain and metabolism simultaneously.
2. TRPA1 (Transient Receptor Potential Ankyrin 1): Often called the “wasabi receptor,” TRPA1 is expressed in sensory neurons and immune cells and is a major sensor of oxidative stress and electrophilic inflammatory mediators. It is activated by reactive aldehydes (like acrolein), H_2_O_2_, and products of lipid peroxidation effectively making it a cellular redox sensor [63]. TRPA1 activation leads to intense pain (e.g., in inflammatory pain conditions) and triggers neurogenic inflammation via release of CGRP and substance P from nerves [65]. In a metabolic context, TRPA1 appears to link obesity-induced oxidative stress to insulin secretion and inflammation. Pancreatic β-cells express TRPA1, and activation by oxidative stress products can stimulate insulin release acutely [63,66]. However, chronic activation (as in metabolic syndrome with constant oxidative stress) may contribute to β-cell dysfunction and inflammation. Additionally, TRPA1 in vagal afferents can influence appetite and inflammation.
  i. SCG bioactives and TRPA1: Given TRPA1’s sensitivity to ROS and electrophiles, the antioxidant constituents of SCGs can significantly impact TRPA1 activity. By scavenging reactive carbonyls and peroxides, SCG polyphenols likely prevent inappropriate TRPA1 activation in tissues during high oxidative stress [63,67,68]. This leads to less pain signaling from TRPA1-containing nerves (beneficial in inflammatory conditions like arthritis or neuropathy). Also, it could protect pancreatic β-cells from oxidative overstimulation. Interestingly, some dietary compounds directly activate or block TRPA1: for instance, cinnamaldehyde (from cinnamon) is a TRPA1 agonist that paradoxically improved insulin sensitivity in mice with obesity by enhancing postprandial insulin via TRPA1 then desensitizing it. SCGs contain cinnamaldehyde-like phenolics (structurally, some feruloyl compounds might weakly agonize TRPA1). If present, these could mimic the effect of a transient TRPA1 activation to boost insulin, followed by desensitization that protects against further oxidative activation (a form of antioxidant gating of TRPA1). Moreover, SCFAs (notably acetate) from SCG fermentation can activate vagal afferents that express TRPA1, possibly modulating satiety and inflammation indirectly. Summing up, SCG compounds likely reduce TRPA1-mediated chronic inflammation (through antioxidant action) while preserving its acute beneficial roles (like glucose-stimulated insulin release). This dual action breaks the link between oxidative stress and TRPA1-driven inflammation, a crucial link in conditions such as diabetic neuropathy and airway inflammation [63,69].
3. TRPM2 (Transient Receptor Potential Melastatin 2): A calcium-permeable channel expressed in immune cells (macrophages, neutrophils, microglia) that is gated by intracellular ADP-ribose and strongly potentiated by oxidative stress (H_2_O_2_) [49]. TRPM2 serves as a sensor coupling ROS levels to Ca^2+^ influx in immune cells. When activated under oxidative bursts, TRPM2 allows Ca^2+^ entry that can trigger cytokine production (e.g., IL-8, IL-6) and chemotaxis. It has been implicated in the regulation of macrophage polarization and the NLRP3 inflammasome: ROS-mediated TRPM2 activation can promote the release of IL-1β, but interestingly TRPM2 also has feedback roles that can limit inflammation by inducing apoptosis or suppressing excessive ROS production [48,70]. In metabolic disease, TRPM2 is important in insulin secretion (β-cells also have TRPM2, linking glucose metabolism to Ca^2+^ signals) and in the immune responses in adipose tissue.
  i. SCG bioactives and TRPM2: By controlling ROS, SCG antioxidants effectively control the “on/off” switch of TRPM2. As mentioned earlier, SCG polyphenols keep ROS below the threshold needed to massively activate TRPM2, thereby preventing overproduction of inflammatory cytokines in tissues like the vascular endothelium and lungs [48,71]. This could translate to less vascular inflammation (TRPM2 in endothelium contributes to damage in high-glucose conditions) and reduced neuroinflammation (microglial TRPM2 drives pathologic cytokine release in oxidative stress). Some evidence suggests that deleting or blocking TRPM2 in models of metabolic inflammation reduces pro-inflammatory cytokine release and insulin resistance, reinforcing that its moderation is beneficial. Thus, SCG compounds dampen TRPM2 activity to an optimal level, enough to allow normal immune signaling but not so much as to provoke chronic inflammation. In doing so, they break a vicious cycle: metabolic stress → ROS → TRPM2 → inflammatory cytokines → more ROS. Notably, one study showed that polyphenols can directly inhibit TRPM2 currents in a neutrophil cell line, though the mechanism is unclear (possibly via scavenging the ADP-ribose generation or interfering with channel gating). Additionally, SCFAs like butyrate might reduce TRPM2 expression in colon macrophages by epigenetic means, complementing the direct antioxidant effects.

In summary, TRP channels serve as critical integrators of oxidative and inflammatory signals in chronic diseases (they sense the “lifestyle” stressors diet metabolites, ROS, thermal changes). Spent coffee ground bioactives modulate TRP channels by (a) preventing their pathological activation through antioxidant and desensitizing effects, and (b) fine-tuning their activity to support beneficial signaling (like metabolic reflexes, insulin secretion, pain control). This TRP-targeted action of SCGs adds a novel dimension to their nutraceutical value beyond chemical antioxidant capacity; they influence the ion channel sensors that orchestrate inflammation and metabolism.

### 4.3. Potassium Channels: Polarizing Immune Responses

Potassium (K^+^) channels in immune cells play a supportive yet essential role: by controlling the membrane potential, they influence Ca^2+^ influx and cell activation. Two types of K^+^ channels are particularly relevant to immunometabolic function: voltage-gated Kv1.3 channels in T lymphocytes and ATP-sensitive K^+^ (KATP) channels in various cells (including macrophages and pancreatic β-cells) [72,73].

Kv1.3 (Voltage-Gated K^+^ Channel): Kv1.3 is highly expressed in effector memory T cells (the pro-inflammatory T cells involved in autoimmune reactions and chronic inflammation). When a T cell is activated, Kv1.3 opens to allow K^+^ efflux, which helps maintain a negative membrane potential. This hyperpolarization is crucial because it provides the driving force for sustained Ca^2+^ entry through CRAC channels. In essence, Kv1.3 keeps Ca^2+^ flowing, thereby enabling full T cell activation (NFAT-driven cytokine production, proliferation). If Kv1.3 is blocked, the T cell membrane depolarizes, Ca^2+^ influx is reduced, and the T cell’s activation program is dampened. This is why Kv1.3 blockers are considered immunosuppressive and are being explored for autoimmune diseases like multiple sclerosis and type 1 diabetes [74,75,76]. Aside from T cells, Kv1.3 is found in macrophages and adipose tissue-resident lymphocytes, impacting adipose inflammation and possibly systemic metabolic regulation.

SCG Bioactives and Kv1.3: It turns out that certain dietary polyphenols can inhibit Kv1.3. Although not studied specifically in SCGs, analogies from green tea (EGCG) and other polyphenols suggest a similar potential. For instance, quercetin and resveratrol have been reported to block Kv1.3 currents in lymphocytes at micromolar concentrations. SCG phenolics like caffeic and ferulic acid might have a mild Kv1.3 inhibitory effect or could modulate its expression. By inhibiting Kv1.3, SCG compounds would cause a partial depolarization of the T cell membrane, limiting Ca^2+^ influx (as discussed) and thus selectively downregulating overly active T cells. The result would be reduced secretion of IFN-γ, IL-17, and other pro-inflammatory cytokines by effector T cells, while possibly sparing regulatory T cells (which rely less on Kv1.3 and more on KCa3.1 channels) [74,77]. This selective targeting is valuable: it can help quell autoimmune-like inflammation in obesity (where pro-inflammatory T cells infiltrate adipose tissue) without globally suppressing immunity. Indeed, in obesity and T2D, Kv1.3 T cells contribute to adipose inflammation; nutritional Kv1.3 modulation might improve insulin sensitivity by reducing this T cell-mediated inflammation. Additionally, Kv1.3 in pancreatic islets (on infiltrating T cells) is a candidate target to delay type 1 diabetes progression a conceivable benefit for high-coffee-consumption populations. While direct data is limited, the hypothesis is that regular intake of SCG bioactives keeps Kv1.3 activity in check, leading to a shift in immune cell polarization toward a less inflammatory state. In practical terms, one might see lower levels of Th1/Th17 cytokines in circulation and improved markers of inflammation (as observed in some coffee intervention studies) [78,79,80].

KATP Channels (ATP-Sensitive K^+^ Channels): KATP channels are unique in that they link cellular metabolism to electrical activity; they close when ATP is high and open when ATP is low. They are well-known in pancreatic β-cells, where closure of KATP in response to rising ATP (from glucose metabolism) triggers insulin release. But KATP channels are also present in immune cells like macrophages and possibly in adipocytes and neurons of metabolic control centers. Recent research has uncovered that KATP channels in macrophages influence their polarization: blocking KATP with glibenclamide skews macrophages away from a pro-inflammatory (M1) state, whereas activating KATP with a drug like pinacidil promotes the pro-inflammatory state [81]. The logic is that when a macrophage is in a nutrient-rich, ATP-high environment, KATP closes (similar to β-cell), causing depolarization that can inhibit certain signaling pathways and reduce inflammatory gene expression. Conversely, under low ATP (energy stress), KATP opens, hyperpolarizing the cell, which can prolong Ca^2+^ entry and NF-κB activity, hence promoting inflammation. In line with this, vulnerable atherosclerotic plaques show upregulated macrophage KATP channel expression correlated with inflammation [82].

SCG Bioactives and KATP: Spent coffee ground components might modulate macrophage KATP channels indirectly. One possible mechanism is through SCFA signaling: butyrate and propionate can enter macrophages and raise cellular AMP (via *FFAR2/3* signaling or by feeding into metabolism), which could tilt the ATP/ADP ratio and thus influence KATP channel status. If SCFAs from SCG fermentation elevate AMP-kinase activity, they might effectively increase KATP opening. However, beneficially, polyphenols like caffeic acid have been noted to improve cellular metabolic efficiency (more ATP per oxygen in mitochondria) which would close KATP and mitigate inflammatory polarization. Moreover, some studies indicate that the surfactant-like compounds in coffee can accumulate in immune cell membranes and directly affect ion channel behavior. While speculative, it is conceivable that diterpenes or other lipids from SCGs intercalate into macrophage membranes near KATP channels (which comprise Kir6.x subunits and SUR subunits) and alter their sensitivity to nucleotides [83,84,85].

The most concrete effect, though, is seen in pancreatic β-cells: coffee consumption (including decaf) is associated with improved β-cell function and insulin secretion. SCG’s niacin (vitamin B3) content, along with its polyphenols, could preserve β-cell metabolic health, keeping KATP channels functioning normally. In T2D, β-cells often have aberrant KATP activity (due to glucolipotoxicity). SCG compounds, by reducing oxidative and ER stress in β-cells, may maintain the proper ATP signaling to KATP, thereby stabilizing insulin release patterns [86,87].

Additionally, the inflammatory macrophage KATP story ties back to diet: a fiber-rich diet (and SCG fiber can be part of that) yields SCFAs that have been shown to reduce inflammation partly via GPR43. GPR43 activation in macrophages can initiate signaling that mimics a high-ATP state (because it promotes a Warburg-like effect increasing citrate/acetyl-CoA for fatty acid synthesis, which tends to close KATP). This might be a stretch, but it is an intriguing connection—postbiotic metabolites from SCGs lead to KATP modulation that reduces macrophage pro-inflammatory polarization. The observed phenomenon that glibenclamide (a KATP blocker) reduces M1 polarization aligns with what a high-SCG diet might achieve metabolically [81].

In summary, K^+^ channels are gatekeepers of immune cell excitability and activation. SCG bioactives influence these channels in ways that promote resolution of inflammation: Kv1.3 inhibition dampens overactive T cells, and a functional tilt in KATP activity discourages M1 macrophage polarization. Together, these effects contribute to an immune profile that is less prone to chronic inflammation, which is beneficial in metabolic diseases. Table 2 summarizes how SCG-derived compounds modulate the discussed ion channels and their implications for chronic disease, while Table 3 complements this by outlining GPCR-mediated signaling pathways targeted by SCG bioactives, highlighting their roles in immunometabolic regulation and disease outcomes.

## 5. GPCR Modulation by Coffee Ground Bioactives

G-protein-coupled receptors (GPCRs) are master regulators of cell responses to hormones, metabolites, and neurotransmitters. A fascinating aspect of nutritional therapeutics is the ability of diet-derived compounds to act as ligands or modulators of GPCRs, thus influencing signaling cascades [98]. Spent coffee grounds yield several metabolites that can engage GPCRs involved in inflammation and metabolism. In particular, SCG bioactives target anti-inflammatory and metabolic GPCRs such as the free fatty acid receptors and adenosine receptors [99,100]. Moreover, there is an intricate crosstalk between GPCR signaling and ion channels (as alluded to earlier: GPCR activation often leads to Ca^2+^ mobilization, and ion flux can feed back to desensitize GPCRs). SCG compounds, by modulating both GPCRs and ion channels, orchestrate a balanced signaling output that promotes immune tolerance and metabolic homeostasis.

### 5.1. Anti-Inflammatory GPCRs: GPR120, GPR43, and Adenosine Receptors

**Figure 4:**
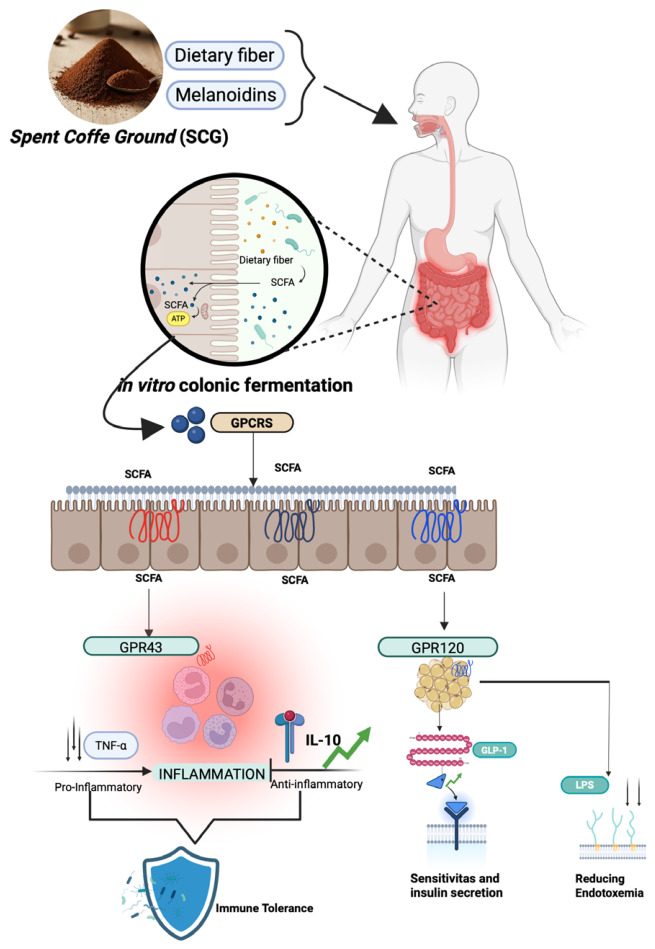
GPCR-mediated immunometabolic effects of spent coffee ground bioactives. Fermentation of SCG dietary fiber and melanoidins by gut microbiota generates SCFAs that activate anti-inflammatory GPCRs, including GPR43 and GPR120. This gut–immune–metabolic axis promotes immune tolerance, improves insulin sensitivity, enhances incretin signaling, and mitigates systemic inflammation associated with metabolic disorders. Created in Biorender. Fahrul Nurkolis. (2026). https://biorender.com (accessed on 18 April 2026).

GPR120 (FFA4): GPR120 is a receptor for long-chain unsaturated fatty acids (like omega-3s) and is predominantly expressed on macrophages and adipocytes. Activation of GPR120 has a strongly anti-inflammatory effect; it inhibits NF-κB and inflammasome activation in macrophages and improves insulin signaling in adipose tissue [88]. It is sometimes called a “metabolic taste receptor” because it senses dietary fat and triggers anti-diabetic pathways (e.g., enhancing insulin sensitizing adipokines). SCGs contain lipids with a significant unsaturated component; for example, linoleic acid constitutes ~43–50% of SCG oil [12]. SCG-derived unsaturated fatty acids can serve as ligands for GPR120 (Figure 4). Indeed, when SCG oil is extracted, it could activate GPR120 similarly to fish oil fatty acids. By engaging GPR120 on tissue macrophages, these fatty acids drive an M2 (anti-inflammatory) polarization and promote secretion of IL-10 while suppressing TNF-α [63]. In obese adipose tissue, GPR120 activation by SCG lipids would reduce crown-like structure inflammation and improve insulin sensitivity of adipocytes (via increased GLUT4 translocation and adiponectin). Additionally, GPR120 on intestinal L-cells, when activated, stimulates GLP-1 release aiding glycemic control. Notably, coffee’s diterpenes and phenolics might indirectly potentiate GPR120 signaling; a recent study suggested cafestol can synergize with omega-3 in macrophages to enhance anti-inflammatory gene expression (the mechanism is unclear but might involve GPR120 crosstalk). Overall, SCG provides “endogenous” GPR120 agonists in the form of its fatty acids, contributing to the anti-inflammatory milieu.

GPR43 (FFA2): GPR43 is a receptor for short-chain fatty acids (SCFAs), especially acetate and propionate, produced by gut fermentation of fibers. Activation of GPR43 has immunomodulatory effects: it has been shown to reduce inflammation by inhibiting NF-κB in certain contexts, although it can also promote neutrophil recruitment acutely [89]. In metabolic terms, GPR43 signaling in adipose tissue helps prevent excessive fat accumulation by increasing energy expenditure and reducing insulin signaling in adipocytes (thereby directing energy to muscles). The SCFAs generated from SCG melanoidin and fiber fermentation (notably acetate and butyrate) are prime activators of GPR43 on colonic epithelial cells and immune cells in the gut (Figure 4) [21]. SCG acts as a prebiotic: its fermentation elevates luminal SCFAs, which then engage GPR43 on gut macrophages to induce secretion of IL-18 and mucosal-healing cytokines, reinforcing the gut barrier and reducing systemic endotoxemia. Moreover, GPR43 on neutrophils when activated can cause a more controlled recruitment (some studies show GPR43 KO mice have exacerbated inflammation in models of colitis and arthritis, indicating GPR43’s protective role) [90]. There is also evidence that GPR43 activation on pancreatic β-cells enhances insulin secretion in response to FFAs, especially in high-fat-diet conditions [91]. Thus, SCG fermentation products via GPR43 could improve insulin dynamics and temper inflammation simultaneously. Interestingly, a nature article reported that maternal SCFA deficiency (thus low GPR43 signaling) led to more inflammation and insulin resistance in offspring, underlining GPR43’s importance [89]. Therefore, a diet including SCG (which boosts SCFA) might mitigate metabolic inflammation through GPR43 contributing to improved glucose homeostasis and lower inflammatory tone in tissues like liver and adipose [92].

Adenosine Receptors (especially A_2_A): Adenosine is a purine nucleoside that modulates inflammation and metabolism through its receptors. The A_2_A adenosine receptor is expressed on many immune cells (T cells, dendritic cells, macrophages) and generally mediates anti-inflammatory effects when activated; for example, adenosine A_2_A stimulation increases cAMP in macrophages, inhibiting TNF-α and IL-12 while promoting IL-10. In the central nervous system, A_2_A on microglia suppresses their pro-inflammatory activation [93,94]. Coffee is famously associated with adenosine receptor antagonism due to caffeine. However, there is a paradox: coffee consumption is linked to anti-inflammatory outcomes, yet caffeine blocks A_2_A which is anti-inflammatory. This paradox is resolved by considering dose, timing, and other coffee components. Acute caffeine blocks A_2_A and can temporarily heighten immune cell activity (useful for pathogen defense or vigilance). Chronic caffeine use, however, leads to upregulation of adenosine receptors and potentially a net anti-inflammatory effect when caffeine is not present. Moreover, other coffee components may activate adenosine pathways: for instance, SCG has small amounts of adenosine itself and related nucleosides that could activate A_2_A (spent grounds from espresso contain soluble nucleosides). Melanoidins might bind adenosine or influence its metabolism. Importantly, SCG diterpenes have been found to have adenosine-like effects in some contexts; for example, cafestol can increase adiponectin and might work via PPAR which crosstalks with A_1_ receptor signaling [95,96].

In immune cells, biased agonism by coffee compounds could occur. Perhaps SCG phenolics act as partial agonists at A_2_A receptors providing enough stimulation to induce an immunotolerant state without full cAMP spike. Meanwhile, caffeine’s presence ensures that overly high adenosine (during stress) does not cause immunosuppression or lethargy. Essentially, coffee bioactives might fine-tune adenosine receptor signaling: when adenosine levels are low, phenolics keep A_2_A mildly active; when adenosine surges (e.g., during ischemia), caffeine tempers the response to avoid excessive suppression. The outcome is a balanced anti-inflammatory effect. In the brain, this is reflected in epidemiologic studies: moderate coffee intake is associated with lower risk of neuroinflammatory diseases (like Parkinson’s and Alzheimer’s) partly attributed to A_2_A blockade on microglia by caffeine reducing chronic inflammation and neurotoxicity. However, it also preserves microglial function in clearing debris (since some activation remains). On the vascular endothelium, A_2_B receptors (which cause inflammation when overactive in high glucose) might be antagonized by caffeine, improving outcomes in diabetes [26,96,97].

In summary, SCG bioactives target multiple GPCRs that naturally resolve inflammation: GPR120 and GPR43 are activated by SCG-derived lipids and SCFAs respectively, leading to systemic anti-inflammatory and metabolic benefits, and adenosine pathways are modulated to favor an anti-inflammatory state without immunodeficiency. The notion of “biased agonism” is apt; these dietary compounds might not act as full agonists, but they bias receptor signaling towards protective pathways (for instance, GPR120 can signal via β-arrestin to inhibit NF-κB, and coffee phenolics may favor the β-arrestin route over Gq).

### 5.2. Crosstalk: Ion Channel GPCR Signaling for Immune Tolerance

**Figure 5:**
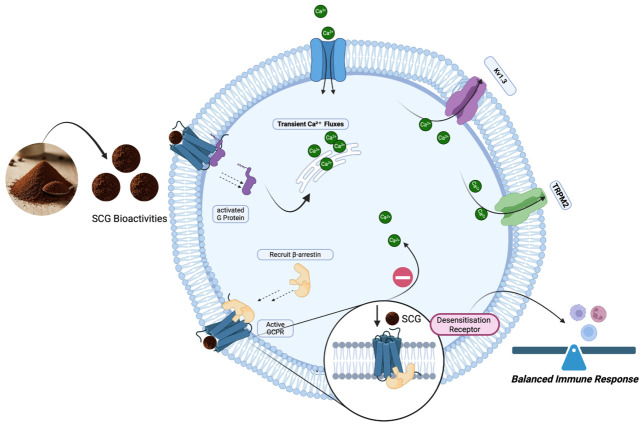
Coordinated ion channel–GPCR crosstalk underlying immune tolerance induced by spent coffee ground bioactives. SCG-derived metabolites initiate GPCR signaling that triggers controlled Ca^2+^ fluxes, while concurrent modulation of ion channels accelerates signal resolution and receptor desensitization. This integrated membrane signaling framework promotes immune tolerance and prevents chronic inflammatory activation characteristic of lifestyle-related diseases. Created in Biorender. Fahrul Nurkolis. (2026). https://biorender.com (accessed on 18 April 2026).

A highlight of this review is the synergistic interplay between the membrane ion channels and GPCRs targeted by SCG bioactives (Figure 5). Ion channels and GPCRs rarely operate in isolation; their signaling pathways intersect at multiple nodes. GPCR activation often leads to Ca^2+^ mobilization (through IP_3_-mediated ER release or store-operated Ca^2+^ entry as GPCR Gq triggers), which we know can activate or modulate ion channels. Conversely, ion channel activity (ion fluxes) can feed back to regulate GPCR responsiveness (via mechanisms like G protein receptor kinase (GRK) activation, β-arrestin recruitment, or membrane potential changes affecting GPCR conformations). SCG bioactives, by co-regulating both, help establish an immune cell signaling milieu that avoids extremes preventing overactivation but also preventing complete anergy.

To illustrate this crosstalk, we propose an integrative mechanistic model centered on the T cell, drawing on individually validated signaling components. It should be noted that while each element of this model is supported by experimental evidence in isolation, the complete integrated sequence has not been validated in a single experimental system and therefore represents a mechanistically grounded but hypothetical framework warranting direct empirical testing. In this model, an SCFA derived from SCG fermentation (e.g., acetate or propionate) activates GPR43 on a T cell, initiating a Gq cascade that promotes IP_3_-mediated Ca^2+^ release from the ER, a well-established signaling event [101]. Under conditions of full immune activation, this Ca^2+^ signal would engage calcineurin and NFAT, and drive pro-inflammatory cytokine transcription. But simultaneously, CGAs from SCGs have depolarized the cell slightly by inhibiting Kv1.3, a mechanism supported by studies of structurally analogous polyphenols including quercetin and EGCG [74,77] and reduced overall ROS. The depolarization means the CRAC channels are not going to conduct as much additional Ca^2+^. So, the Ca^2+^ signal from GPR43 is kept moderate. Moderate Ca^2+^ will preferentially activate CaMK pathways and perhaps induce anergy/tolerance genes rather than full effector cytokines. Additionally, Ca^2+^ activates GRKs that phosphorylate the GPR43, promoting β-arrestin recruitment and receptor desensitization [101]. Interestingly, β-arrestin recruitment to GPR43 in macrophages is known to bias signaling toward anti-inflammatory outcomes (like inducing IL-10). Thus, the initial GPCR activation triggered its own brake via Ca^2+^/GRK and SCG’s ion channel effects enhanced that brake by adding more Ca^2+^ regulatory feedback and lowering the risk of reactivation (ion channels now in refractory state). In essence, ion channel modulation accelerates GPCR desensitization/tolerance. This is beneficial in chronic disease where continuous GPCR stimulation (by inflammatory mediators or metabolic ligands) would otherwise keep immune cells in a pro-inflammatory state. SCG bioactives help immune receptors “cool off” after initial activation.

Adenosine A_2_A receptor on a neutrophil gets activated (say by adenosine from cells or by a coffee metabolite). This triggers cAMP which can activate K^+^ channels (like KCa3.1) hyperpolarizing the cell. The hyperpolarization enhances Ca^2+^ influx through any open CRAC or P2X channels. But because the neutrophil is also experiencing antioxidant effects from SCGs, its NADPH oxidase is toned down, producing less ROS to sustain P2X7 opening [102]. This attenuation of NADPH oxidase-derived ROS limiting P2X7-mediated inflammatory signaling represents an experimentally supported mechanism, independent of its effects on chemotaxis [102]. The transient Ca^2+^ rise from hyperpolarization triggers the neutrophil to go into a migratory but less inflammatory mode (some Ca^2+^ is needed for chemotaxis, but not too much for NETosis). Then, β-arrestin comes to the A_2_A receptor due to PKA phosphorylation and the neutrophil stops responding to adenosine, preventing excessive suppression of microbicidal activity. This fine balance ensures the neutrophil can fight infection but not cause tissue damage by overshooting inflammation.

From a systems perspective, SCG compounds induce a pattern of repeated mild stimulation and quick desensitization of GPCRs coupled with stabilization of ion homeostasis. This likely manifests as improved immune tolerance as immune cells respond to dangerous stimuli but then quickly return to baseline rather than staying chronically activated. Likewise, in metabolic tissues, hormones and neurotransmitters signal appropriately without causing receptor resistance (e.g., insulin or leptin resistance can be thought of as a kind of GPCR/RTK desensitization due to chronic stimulation; coffee consumption is linked with improved insulin and leptin sensitivity, possibly through reducing chronic stimulatory tone via its effects on inflammation and maybe adenosine signaling). This concept parallels well-characterized physiological phenomena such as the post-prandial anti-inflammatory rebound and exercise-induced immune modulation, both of which involve transient GPCR and ion channel activation followed by resolution, processes epidemiologically associated with reduced chronic disease risk [2,8]. The difference is that SCG bioactives may pharmacologically recapitulate these transient activation–resolution cycles on a sustained dietary basis.

Notably, hardly any prior reviews have connected SCG bioactives to ion channel GPCR crosstalk in a systemic fashion. We are proposing that the net effect of SCG bioactives is immune homeostasis through coordinated membrane signaling modulation. By activating GPCRs that initiate anti-inflammatory programs (which involve transient Ca^2+^ signals and cAMP), and simultaneously ensuring those signals do not run away (via ion channel control and promotion of desensitization), SCG compounds achieve an immune “tolerance” state. This is not tolerance in the sense of immunosuppression, but rather the healthy resolution phase of immune responses akin to what happens after meal ingestion or exercise (transient inflammation followed by anti-inflammatory rebound).

One could describe it as inducing the cellular equivalent of a “coffee break”: a brief activation followed by a calm period, which ultimately increases efficiency and prevents burnout. For metabolic cells, this might mean better rhythmic insulin release and prevention of receptor downregulation. For immune cells, it means readiness to respond, but avoidance of chronic inflammatory signaling.

In conclusion, SCG bioactives exemplify a new class of nutraceutical compounds that do not hit one target hard (as a drug might) but gently modulate multiple membrane targets to orchestrate a beneficial adaptive response. The interplay of GPCR activation and ion channel feedback fostered by SCGs could underlie observed epidemiological benefits of coffee in inflammatory and metabolic disorders. This holistic mechanism situates SCG as an ideal candidate for next-generation immunonutrition, where the goal is to retrain and balance the immune–metabolic interface, not simply block it.

## 6. Immunomodulatory Implications in Chronic Diseases

The combined antioxidant, ion channel-modulating, and GPCR-targeting actions of spent coffee ground bioactives translate into tangible benefits across a spectrum of chronic, lifestyle-related diseases. In these conditions, reducing chronic inflammation (metaflammation), restoring redox homeostasis, and improving cell signaling can ameliorate pathology. We highlight how SCG-derived compounds could impact two major categories: metabolic disorders (T2D and obesity) and inflammatory disorders impacting cardiovascular and neurodegenerative health.

### 6.1. T2D and Obesity: Immune–Metabolic Modulation

T2D and obesity are paradigmatic immune–metabolic diseases. They are characterized by insulin resistance, ectopic fat accumulation, and chronic “metaflammation”, an ongoing, low-grade inflammatory state driven largely by nutrient excess and adipose tissue dysfunction [2,103]. Macrophages infiltrate adipose tissue (shifting from M2 to pro-inflammatory M1), T cells produce excess IFN-γ and IL-17, and adipocytes release inflammatory adipokines. This immune activation feeds into insulin resistance (e.g., TNF-α and IL-6 inhibit insulin signaling pathways) [1,104]. Additionally, pancreatic β-cells are under inflammatory and oxidative attack, leading to impaired insulin secretion. The membrane signaling disturbances include overactive NF-κB, NLRP3 inflammasome activation, and possibly dysregulated ion channels like KATP in β-cells or Kv1.3 in islet-infiltrating T cells.

Reduced Metaflammation: SCG bioactives can break the cycle of metabolic inflammation. By polarizing macrophages back towards an anti-inflammatory state (via GPR120 activation and KATP modulation) and by inhibiting pro-inflammatory T cell activity (via Kv1.3 and adenosine pathways), SCG compounds lower tissue inflammation. For instance, in mice with obesity, dietary polyphenols from coffee have been shown to reduce macrophage infiltration and inflammatory gene expression in adipose tissue. The SCG components likely responsible include CGAs and its metabolites, which suppress NF-κB and JNK signaling in adipocytes and macrophages. The net result is higher adiponectin and lower TNF-α levels in adipose tissue, an environment more conducive to insulin action [63,104]. Indeed, coffee consumption in epidemiological studies is associated with reduced circulating markers like CRP and IL-6, indicating less systemic inflammation. Our mechanistic exploration suggests SCG’s effect on ion channels (like inhibiting TRPA1’s oxidative activation and TRPM2’s inflammasome signal) is key in achieving this anti-inflammatory state in metabolic tissues.

Improved Insulin Signaling: A less inflamed environment directly translates to better insulin signaling, as cytokine suppression relieves IRS-1 and AKT from inhibition. But SCG compounds also have direct insulin-sensitizing actions. CGAs has been shown to improve insulin sensitivity in vivo, partly by inhibiting glucose-6-phosphatase in the liver (reducing gluconeogenesis) and partly by enhancing muscle glucose uptake. Additionally, the SCFA propionate from SCG fermentation increases GLP-1 secretion and activates GPR43 on adipocytes, which has been linked to improved insulin sensitivity and adipose browning [92,105]. GPR43 activation in adipocytes prevents hypertrophy and insulin resistance in obesity models [89,90]. Thus, SCG via gut fermentation can recapitulate some benefits of dietary fiber on insulin action, a crucial point since fiber is often lacking in calorically rich diets. Furthermore, the minor compounds like niacin in SCG (coffee is a notable niacin source post-roasting) can improve lipid profiles and adiponectin, indirectly aiding insulin sensitivity.

β-cell Ca^2+^ Protection: Pancreatic β-cells in T2D suffer from chronic high glucose and fatty acid exposure, leading to exaggerated Ca^2+^ oscillations and eventual apoptosis. SCG bioactives can protect β-cells in several ways. First, the antioxidant effect reduces glucotoxicity and lipotoxicity; less ROS means less activation of stress kinases that impair insulin granule exocytosis. Second, by modulating KATP and TRPV1, SCG compounds help normalize β-cell excitability [106,107]. For example, caffeine’s presence might improve the efficiency of glucose-induced depolarization (some studies show caffeine acutely enhances insulin secretion, through the cAMP/EPAC pathway). More importantly, the sustained intake of coffee components can prevent β-cell overstimulation: if Kv channels or TRPM2 in β-cells are tempered, the cells will not dump insulin at inopportune times and then fatigue. We can infer from a long-term study that decaf coffee consumption was associated with a slower decline in C-peptide levels in people with diabetes, pointing to preservation of β-cell function, likely due to beneficial components like CGA and not caffeine. This fits with our concept that SCG polyphenols guard β-cells by stabilizing Ca^2+^ handling (less ER stress, better mitochondrial function) and reducing islet inflammation (fewer infiltrating immune cells and cytokines, partly because those immune cells have been kept in check by SCG’s systemic effects). In essence, SCG bioactives create a healthier islet microenvironment: more regulatory immune cells (like perhaps more IL-10 producing macrophages given GPR120 activation) and less oxidative damage, allowing β-cells to maintain insulin output longer.

Human evidence corroborates these mechanistic insights: habitual coffee intake is associated with a lower risk of developing T2D (each additional cup per day gives ~6–9% risk reduction) [108,109]. Interventional studies found improved glucose tolerance and insulin sensitivity with high-polyphenol coffee consumption. Spent coffee grounds, being an even richer source of many of these compounds, could be formulated into functional foods or supplements for metabolic syndrome patients. For overweight individuals, incorporating SCGs (e.g., as flour in bakery products or as a fermented drink) might reduce chronic inflammation (lowering adipose MCP-1 and plasma CRP) and modestly improve insulin responsiveness.

Additionally, weight management could benefit: SCG’s fiber content promotes satiety and its SCFAs promote fat oxidation. There is a precedent in a study where overweight adults consuming a coffee polyphenol/fiber mixture had reductions in visceral fat over 12 weeks. SCGs likely can mirror that effect, acting as a sustainable slimming ingredient that addresses the inflammatory component of obesity too.

### 6.2. Cardiovascular and Neuroinflammatory Disorders: Cellular Homeostasis Restoration

Chronic inflammatory processes also underlie many cardiovascular diseases (like atherosclerosis, hypertension) and neurodegenerative diseases. Lifestyle factors (diet, coffee consumption, etc.) have measurable effects on these conditions. The bioactives in SCGs, through their membrane-centric actions, can confer protection in these realms as well.

Cardiovascular Disease (CVD): Atherosclerosis is now known to be driven by inflammation of the arterial wall macrophages that ingest lipids and become foam cells, secreting cytokines that further plaque development. There is also oxidative stress from lipoprotein oxidation [110]. SCG compounds can intervene at multiple points: their antioxidant action prevents LDL oxidation (chlorogenic and ferulic acids from SCGs were shown to inhibit copper-induced LDL oxidation in vitro). If less oxidized LDL forms, macrophage uptake is reduced and the inflammatory cascade blunted. Moreover, SCG’s impact on endothelial Ca^2+^ stability is crucial. Endothelial dysfunction in early atherosclerosis is characterized by excess ROS and decreased nitric oxide (NO) due to, e.g., uncoupled eNOS. CGA has demonstrated that it can improve endothelial function by reducing vascular NADPH oxidase-derived ROS and increasing NO bioavailability [30,111]. This translates to better vasodilation and lower blood pressure; indeed, hypertensive rats given CGAs show significant BP reduction [31]. If we extend that to humans, SCG supplements (rich in CGA) might help manage hypertension. The Ca^2+^-NO coupling in endothelium is interesting: normally, when intracellular Ca^2+^ rises, eNOS produces NO. In oxidative conditions, Ca^2+^ can instead trigger contraction or an anti-NO effect. By lowering oxidative tone, SCGs ensure that endothelial Ca^2+^ signals lead to vasodilation (via NO) rather than vasoconstriction (via Ca^2+^-mediated smooth muscle contraction). Additionally, potassium channel modulation (like improving K_Ca_ channel function on endothelium) by SCG polyphenols aids endothelium-derived hyperpolarizing factor (EDHF) responses, giving further vasoprotective effect.

Inflammation in vessels is also mitigated: GPR120 on macrophages in plaques, when activated by SCG unsaturated fatty acids, inhibits the secretion of MMPs and pro-inflammatory cytokines, potentially stabilizing plaques and preventing their rupture. GPR43 activation by SCFAs may reduce adhesion molecule expression on endothelium, lowering monocyte recruitment to plaques [89]. There is evidence that mice lacking GPR43 have worse high-fat-diet-induced arterial inflammation, implying SCFAs (like those from SCGs) through GPR43 protect arteries [90].

Epidemiologically, moderate coffee consumption is associated with lower risk of coronary heart disease and stroke. Some large studies have found U-shaped relationships, with the lowest risk around 3–4 cups/day. The mechanisms we have described (improved endothelial function, reduced inflammation, better lipid metabolism due to polyphenols boosting liver AMPK) all support these observations. SCGs could be thought of as concentrated heart-healthy compounds. One could envision adding SCG extract to a heart patient’s diet to provide extra CGAs (for BP and vascular health) and melanoidins (for gut-derived benefits impacting cholesterol metabolism, since melanoidins can bind bile acids and reduce cholesterol levels) [112,113].

Neuroinflammatory Disorders: Chronic neuroinflammation is a feature of neurodegenerative diseases like Alzheimer’s and Parkinson’s disease (AD, PD), as well as contributing to depression and cognitive decline in aging [114]. Microglia, the brain’s immune cells, when persistently activated, release cytokines and ROS that damage neurons. Coffee consumption has been linked to lower risk of PD and possibly slower cognitive decline; much of this has been attributed to caffeine’s effect on adenosine A_2_A receptors in the brain [115,116]. However, other coffee constituents cross the blood–brain barrier too (ferulic acid, for instance, can penetrate and has been shown to have anti-inflammatory effects in the brain, including reducing amyloid-induced microglial activation). SCG phenolics and their metabolites (like phenylpropionic acids from gut metabolism of melanoidins) likely enter circulation and reach the brain, where they can exert antioxidant and anti-inflammatory influence.

Microglial GPCR Regulation: Microglia express adenosine receptors (A_2_A and A_3_ notably). A chronic blockade of A_2_A by caffeine is considered neuroprotective in PD, as it prevents microglial overactivation that would produce neurotoxic cytokines. SCG’s caffeine contributes to this. But interestingly, microglia also have GPR43 (since SCFAs can modulate brain inflammation via the vagus nerve, directly crossing the BBB in small amounts). A study showed that SCFA depletion in mice led to microglial dysfunction and exaggerated neuroinflammation; restoring SCFAs improved microglial homeostasis [89,116,117]. Thus, SCG fermentation products (SCFAs) might help maintain microglia in a “surveying” non-inflammatory state, possibly through GPR43 and GPR41 in the CNS or via indirect systemic anti-inflammatory signals. Additionally, TRPA1 on sensory neurons has been implicated in migraine; coffee’s ability to reduce pain might partly be via TRPA1 modulation, preventing cortical spreading and depression triggers from oxidative stress.

One remarkable demonstration of coffee’s neuroprotective effects is in a mouse model of Alzheimer’s, where coffee polyphenol extract reduced brain amyloid and microglial activation, accompanied by improved memory. SCG contains the same polyphenols, so could be even more potent. Melanoidins, in particular, might exert benefits in the gut–brain axis: by fostering a healthy microbiome and producing butyrate, they reduce systemic inflammation, which is increasingly recognized as a driver of neurodegeneration. Lower peripheral inflammation leads to lower neuroinflammation (via reduced trafficking of inflammatory monocytes to the brain and lowered inflammatory cytokines that can penetrate the BBB) [22,100,118].

Endothelial Ca^2+^ Stability (Neurovascular): Many neuro disorders involve impaired blood flow or BBB leakage. Endothelial cells of the BBB benefit from the same improvements as peripheral endothelium via SCG compounds, better NO production, lower oxidative stress, and thus improved cerebral perfusion [119]. This might explain why coffee drinkers have lower incidence of stroke; SCG compounds like ferulic acid are known to reduce infarct volume when given to rodents after a stroke (owing to antioxidant and anti-excitotoxic effects).

In summary, from heart to brain, SCG bioactives contribute to restoring homeostasis at the cellular level: oxidative stress is mitigated, inflammatory signaling dampened, and normal cell signaling (whether it is insulin, NO, or neurotransmitter) is maintained. Lifestyle-related chronic diseases share a theme of being stuck in a vicious cycle of stress and maladaptive signaling; SCG components help break those cycles.

## 7. Translational and Sustainability Perspective

The potential of spent coffee grounds as a health-promoting resource goes hand-in-hand with a vision for sustainable nutrition. Rather than treating SCGs as waste, we can upcycle it into value-added products that support health (a concept aligning with both precision nutrition and environmental conservation). In implementing SCGs for human use, however, practical considerations like safety, standardization, and regulatory compliance arise.

### 7.1. From Coffee Waste to Precision Nutrition

Exploiting SCGs for health requires innovative approaches to integrate it into diets. One avenue is developing functional food ingredients from SCGs. For example, SCGs can be dried, milled into a high-fiber, polyphenol-rich flour and incorporated into baked goods, nutrition bars, or smoothies. This not only adds antioxidant content but also increases dietary fiber (SCG is ~50% dietary fiber by dry mass, including cellulose and hemicelluloses) [12,99,120]. Early trials have shown that baked products with 5–10% SCG flour are sensorially acceptable and have higher antioxidant activity and improved glycemic response (due to fiber slowing carbohydrate absorption). Such products could target individuals with metabolic syndrome providing satiety (fiber), better postprandial glucose control, and anti-inflammatory benefits in one package.

Another exciting frontier is postbiotic formulations. Instead of (or in addition to) consuming SCGs directly, one could ferment SCGs with specific microbial strains to generate concentrated bioactive metabolites. We discussed solid-state fermentation yielding enriched polyphenol extracts; liquid fermentation (like kombucha culture fermenting SCGs) can produce beverages containing SCFAs, fermentation-derived B vitamins, and breakdown products of melanoidins that might act as signaling molecules. These postbiotic drinks could serve as “gut tuning” therapies much like how fermented foods confer health benefits beyond their raw ingredients. Since SCG fermentation can be tuned by selecting microbes (e.g., lactobacilli, which might produce more butyrate vs. yeasts that produce more phenolic breakdown), one could tailor the metabolic output for specific conditions. For instance, a SCG kombucha rich in acetate and CGAs derivatives might be ideal for an individual with obesity (acetate for GPR43 in adipose, phenolics for antioxidant effect), whereas one rich in butyrate and peptides might benefit someone with ulcerative colitis (improving colonic regulatory T cells and barrier function). This concept aligns with emerging research by some groups aiming to create synbiotic therapies (prebiotic + probiotic = postbiotic) from SCGs.

Moreover, SCGs could be harnessed in encapsulated supplement form. Given its high content of soluble antioxidants, an SCG extract (water or ethanol extract) could be encapsulated and standardized to a certain CGA or melanoidin content. This would be akin to green coffee bean extract supplements, but potentially broader in spectrum (including the unique melanoidin polymer antioxidants). Such a supplement might appeal to those who want the benefits of coffee’s bioactives without the caffeine or for those who do not consume coffee. It could also be paired with existing treatments: for example, an SCG extract with GPR120 activators could complement omega-3 fish oil therapy in an anti-inflammatory regimen, perhaps enhancing it (since coffee diterpenes plus omega-3 might synergistically activate GPR120 more than fish oil alone).

Precision nutrition comes into play by matching SCG-derived interventions to individuals’ genetic and microbiome profiles. As we understand that certain GPCR polymorphisms (say a variant in GPR43 or adenosine receptors) might make one person respond differently to SCG compounds, we could tailor the approach [121,122]. Someone with a loss-of-function GPR43 might not benefit as much from SCFAs; maybe they need higher doses or alternative strategies whereas someone with an overactive NLRP3 might particularly benefit from TRPM2-targeting antioxidants in SCGs. With multi-omics data (genomics, metabolomics), we can predict who will respond best to SCG nutraceuticals.

From a sustainability standpoint, using SCGs in food or supplements closes the loop of coffee production, reducing waste and environmental burden [12]. The coffee industry could see cost savings or even profit by selling SCG-derived products. Small-scale implementations are already seen: SCGs are used to grow mushrooms, to make biofuels, and in cosmetics. Nutraceutical use could be the next big area. Imagine cafes or roasters partnering with food companies to collect SCGs and produce a line of “coffee fiber” snacks or “mocha antioxidant” beverages. This not only appeals to eco-conscious consumers but also to health-conscious ones.

A particularly innovative idea is to incorporate SCGs into functional fermentation systems. For instance, by developing a yogurt or kefir with SCG fiber, the dairy probiotics ferment the SCGs in situ, enhancing the yogurt’s nutritional profile (extra antioxidants, fiber, and new metabolites). Similarly, SCGs could be added to animal feed in agriculture to improve the animals’ gut health and reduce emissions (some studies have given SCGs to cows to cut methane, leveraging its tannins and fiber) [123]. The animals’ products (milk, meat) might then even carry some of the SCG benefits forward (e.g., higher antioxidant content in milk).

In terms of aligning with current trends, SCG utilization touches on multiple UN Sustainable Development Goals: responsible production and consumption (SDG 12), good health and well-being (SDG 3), and climate action via waste reduction (SDG 13). It exemplifies how “one man’s trash can be another man’s treasure”, here, the treasure being improved health.

### 7.2. Safety, Standardization, and Regulatory Outlook

Using SCGs for human consumption requires careful consideration of safety and quality. Fortunately, spent coffee grounds are generally considered safe because they are essentially the by-product of a food (coffee) that is widely consumed. Recent regulatory opinions in the EU have actually treated SCG as not novel if it is compositionally similar to roasted coffee (which it is, minus some water-soluble components) [21,99]. For example, the European Food Safety Authority (EFSA) in a 2022 assessment noted that SCG did not raise safety concerns as an ingredient up to certain intake levels, and it can be introduced as a novel food ingredient after basic toxicological evaluation. One caveat: SCGs can absorb contaminants from the environment (e.g., if coffee beans had mycotoxins or if brewed using contaminated water, those residues might concentrate in SCGs) [124]. Thus, source control and testing are vital. Any SCG used in food or supplements should be tested for mycotoxins (like OTA), acrylamide (formed during roasting; some may be in melanoidins), and heavy metals. Typical analyses so far show levels within safe limits, but it must be ensured batch-to-batch.

Another factor is caffeine content. While SCG has less caffeine than coffee brew, it is not caffeine-free [125]. If one were to eat a lot of SCG-fortified food, caffeine intake could accumulate. However, since SCG tastes quite bitter at high concentrations (due in part to residual caffeine and polyphenols), organoleptic factors naturally limit how much is used. Still, standardizing SCG preparations to a consistent (and possibly low) caffeine content may be needed for supplements (perhaps by water washing the SCG first to decaffeinate it further).

Standardization of bioactive content is a key challenge. SCGs from different coffees and brewing methods vary in composition (e.g., SCGs from espresso vs. drip coffee have different moisture and oil content; Robusta SCG has more caffeine and less oil than Arabica) [12,124]. To develop reliable products, an omics-based standardization approach could be used: profiling batches using metabolomics to quantify key compounds (CGAs, caffeic acid, cafestol, fiber content, etc.) and blending or concentrating to achieve a target specification. For instance, one could set that each 5 g serving of SCG flour provides 500 mg total phenolics (GAE), 50 mg CGAs, and 2 g fiber. Advanced methods like NMR or LC-MS fingerprinting can ensure each batch meets a “fingerprint” associated with efficacy (much like phytopharmaceuticals do). This is feasible given SCG’s complex matrix, and a combination of multiple marker compounds might be used.

On the regulatory front, SCGs would likely fall under existing categories. In the EU, if treated as a novel food, approval is needed but the hurdle is not too high given the food origin. In the US, SCGs could probably be self-affirmed as GRAS (Generally Recognized As Safe) if appropriate toxicology data is compiled (coffee itself is GRAS; SCG contains known coffee components). As mentioned in [21], SCG has been considered not novel in some jurisdictions due to its similarity to conventional coffee. Coffee silverskin (another coffee by-product) was recently authorized as a novel food in Europe, setting a precedent (silverskin is compositionally somewhat like SCGs in fiber and phenolics, though SCG has more melanoidins).

One regulatory consideration is labeling: if we use SCG in foods, it might simply be labeled “coffee dietary fiber” or “coffee ground wholemeal”, etc. In supplements, it could be “Coffee (*Coffea arabica*) spent ground extract, standardized to % polyphenols.”

Safety studies specific to SCGs are still sparse, but given its constituents, we anticipate a few issues. High doses of CGAs can cause laxative effects in some people (and SCG’s high-fiber can too). So, mega-dosing might lead to GI upset. Gradual introduction would mitigate this. Also, those with sensitivity to caffeine should note that SCG products might contain some.

Lastly, ensuring palatability and acceptance is part of practical safety; if something tastes terrible, people might adulterate it (like adding lots of sugar) which could counteract benefits. Thus, recipes and formulations must make SCG-based foods enjoyable are necessary. There is progress here: some studies baked SCGs into cookies and found that at ~5% inclusion, people liked it and it boosted antioxidant levels of the cookies significantly [12]. Consumer education will also help: framing SCG foods as premium, sustainable, and healthful will increase acceptance.

In conclusion, bringing SCGs from waste to nutraceutical requires crossing t’s and dotting i’s on safety and standardization, but none of the challenges seem insurmountable. On the contrary, the momentum of sustainability and wellness trends strongly favor such innovation. The opportunity to simultaneously reduce waste and fight chronic diseases is truly a win–win scenario.

## 8. Future Directions and Conclusions

The exploration of spent coffee grounds as a source of bioactives is a compelling example of marrying sustainability with advanced health science. This review has outlined the multifaceted mechanisms by which SCG compounds can modulate redox status, ion channel activity, and GPCR signaling to combat lifestyle-related chronic diseases. To fully capitalize on this potential, future research should take a multi-disciplinary, multi-omics approach:

1. Multi-omics and Mechanistic Studies: We need integrated studies using genomics, proteomics, metabolomics, and even electrophysiology to map out how SCG compounds act in various cell types. For instance, transcriptomics of macrophages treated with SCG extract could reveal a signature of gene changes (e.g., upregulation of antioxidant genes, suppression of inflammasome components). Proteomics might show increased expression of ion channel regulators or GPCR kinases that align with our proposed crosstalk mechanism. Such comprehensive profiling will validate and possibly lead to the discovery of new targets (maybe SCG affects other channels or receptors we have not considered, like insulin receptors or toll-like receptors). Additionally, patch-clamp studies on immune cells in presence of SCG compounds can directly confirm inhibition of Kv1.3 currents or TRP channel modulation.
2. Personalized Nutrition Angles: As noted, individuals have genetic differences that affect their response to dietary components. One intriguing direction is investigating GPCR polymorphisms (like those in FFARs or adenosine receptors) in relation to coffee/SCG benefits. It could be that certain polymorphisms in GPR120 (which are linked to obesity risk) might be “rescued” by higher intake of SCG unsaturated fats or diterpenes acting on that receptor. This could lead to personalized SCG-based interventions, e.g., a nutraceutical tailored for someone with the *FFAR4* risk variant. Similarly, identifying “responders” vs. “non-responders” to SCG supplementation in a trial based on genotype will advance precision nutraceuticals.
3. Microbiome Research: Since SCG’s fermentation is key to many benefits, future work should delineate which microbial taxa are most important. Does SCG favor the growth of specific beneficial microbes (perhaps *Bifidobacterium* due to melanoidins acting like prebiotics)? And how does that translate to host health? Fecal metagenomic analysis in people with SCG-fortified diets could show enrichment of butyrate-producing bacteria correlating with improved metabolic markers. We might also consider developing synbiotic combinations pairing SCG with probiotic strains known to efficiently ferment it (for example, a *Lactobacillus plantarum* strain that we isolate from a kombucha that loves SCG). This could maximize SCFA output and have beneficial effects.
4. Clinical Trials: Ultimately, interventions need testing in humans. Short-to-medium term clinical trials (6–12 months) on populations at risk for T2D or CVD could test SCG supplementation. Endpoints would include insulin sensitivity (HOMA-IR, glucose tolerance), inflammatory markers (CRP, IL-6, TNF), blood pressure, lipid profiles, and even functional outcomes like endothelial function (via flow-mediated dilation) or cognitive tests (for neuroprotective aspects). Such trials would provide evidence to support SCG as an effective dietary addition. Given coffee itself has many trials and is safe, SCG trials should be straightforward to approve ethically.
5. Product Development and Innovation: Food scientists and chemists should address some practical aspects: how to best extract or formulate SCG for maximal benefit and bioavailability. This could include developing a standardized SCG extract that preserves melanoidin structure (which is huge and not easily taken up) but perhaps breaks it into slightly smaller units that still have activity and can be absorbed to an extent, or encapsulating SCG phenolics in nanoparticles to enhance their tissue targeting (maybe to get more into the brain for neuroinflammation). Another idea is combining SCGs with other synergistic nutraceuticals. Given SCG has a broad array of compounds, it might synergize with, say, turmeric (curcumin) or ginger (gingerol) to cover even more signaling pathways for inflammation. Such combinations could be potent yet still natural.

Several limitations of this review warrant acknowledgment. First, the majority of mechanistic evidence reviewed here derives from in vitro and animal studies; clinical trials specifically investigating SCG bioactives on ion channel and GPCR-mediated pathways in humans remain scarce. Second, the bioavailability of key SCG compounds (particularly bound polyphenols and melanoidins) in vivo is incompletely characterized, and interindividual variation in gut microbiota may substantially modulate the production of bioactive SCG metabolites such as SCFAs. Third, the compositional variability of SCGs, depending on coffee species, roasting degree, and brewing method, limits direct comparability across studies. Fourth, direct evidence linking SCG-specific bioactives (as opposed to coffee bioactives in general) to the ion channel and GPCR mechanisms described herein is largely inferential at this stage. Future well-controlled human intervention trials using standardized SCG preparations are needed to validate the mechanistic hypotheses proposed in this review.

In conclusion, spent coffee grounds embody the concept of “functional waste” a prodigious resource hiding in plain sight. Over the last decade (2015–2025), research has increasingly shown that SCGs are rich in substances that can correct the immune–metabolic imbalances central to many chronic diseases. By targeting ion channels like TRPV1/TRPA1 (sensory–inflammatory reflexes) and Kv1.3/KATP (immune cell activation), and GPCRs like GPR120/GPR43 (inflammatory resolution pathways), as well as providing robust antioxidant support, SCG bioactives tackle the problem from multiple angles simultaneously. This is a more holistic approach compared to single-target drugs, and in chronic, multifactorial diseases, such an approach is likely more effective. Not only do SCGs promise health benefits, but using them contributes to sustainability, reducing the massive waste burden of the coffee industry.

Lifestyle diseases are fueled by immune dysregulation, oxidative stress, and faulty cell signaling. Spent coffee grounds once thrown away turn out to contain a treasure trove of compounds that re-equilibrate these processes. They quell inflammation, buffer oxidative stress, and normalize signaling through membranes. This positions SCG as a prime candidate for the next generation of immunonutrition or “membrane nutrition” interventions. The road ahead will involve fine-tuning the delivery and verifying the benefits in clinical settings, and the prospects are exciting. It is poetic that the literal grounds of our coffee, something that has energized human civilization for centuries, might also help ground our immune system in a state of health. In the broader context, SCGs exemplify how we can find solutions to modern health challenges by looking more closely at what we already have sometimes; the answers to complex problems are lying at the bottom of our coffee cup.

## Figures and Tables

**Table 1:** Major bioactive compounds present in spent coffee grounds and their antioxidant and immunomodulatory effects.

Compound (Class)	Presence in SCG	Antioxidant Mechanisms	Immunomodulatory Effects	References
Chlorogenic acids (CGAs) (Polyphenol)	Abundant (incl. 5-CQA, 3-CQA); partly extracted, partly bound	Potent free radical scavengers; inhibit lipid peroxidation Suppress NADPH oxidase (NOX) activity, reducing ROS generation Chelate metal ions (Fe^2+^), reducing Fenton reactions	Attenuate NF-κB and MAPK activation → lower TNF-α and IL-6 Improve endothelial function via increased NO bioavailability (NOX inhibition) Enhance insulin sensitivity by reducing oxidative stress in metabolic tissues	[12,30,31]
Caffeic & Ferulic acids (Polyphenols)	Present as CGA components and in bound form; released by hydrolysis	Strong ROS scavengers (hydrogen donation to quench radicals) Ferulic acid stabilizes lipid membranes against oxidation ↑ cellular antioxidant enzymes (SOD, GSH peroxidase)	Block NF-κB nuclear translocation → inhibit IL-1β and IL-6 release Caffeic acid reduces iNOS and COX-2 expression → dampens inflammatory NO and PGE_2_ Support M2 macrophage polarization via NRF2 activation	[12]
Melanoidins (Maillard polymers)	High levels (~15–25% of SCG dry mass); polymeric carbohydrates with bound phenolics	“Redox buffering”: scavenge radicals (ABTS, DPPH assays show high activity) Chelate transition metals Resist digestion; release phenolic antioxidants upon gut fermentation Carry antioxidant activity to the colon	Inhibit adhesion of pathogenic bacteria → ↓ gut inflammation Act as prebiotics: fermentation yields SCFAs (acetate, butyrate) → activate GPR43 ↓ colonic pro-inflammatory enzymes; induce apoptosis in colon cancer cells Overall “Maillardized antioxidant dietary fiber” effect tempering local and systemic inflammation	[21,32]
Caffeine (Alkaloid)	Residual levels (0.1–0.4% dry weight); lower than brewed coffee but non-negligible	Weak direct antioxidant (minor ROS scavenging vs. polyphenols) Indirectly protects against oxidative stress via CNS and hormonal effects (e.g., enhancing adrenaline → induces antioxidant enzymes) Synergizes with polyphenols in preserving redox balance	Adenosine A_2_A receptor antagonist → heightens immune surveillance Neuroprotective: A_2_A blockade limits microglial overactivation (caffeine consumers show reduced neuroinflammatory markers) Chronic intake associated with lower IL-1β and inflammasome activation Stimulates adrenaline → via β_2_-adrenergic receptors suppresses TNF-α production Net effect context-dependent; moderate caffeine often correlates with lower CRP	[12,25,26]
Cafestol & Kahweol (Diterpenes)	Present in SCG oil fraction; concentration depends on brew method (higher in espresso/boiled); cafestol > 0.2% and kahweol traces in Arabica SCG	↑ cellular GSH and phase II enzymes Kahweol inhibits inducible NO synthase and downregulates *NOX2*, reducing ROS Both scavenge radical species and protect membranes from oxidation (evidenced in hepatocyte studies)	Cafestol reduces IL-8, MCP-1 and other chemokines in endothelial and immune cells Kahweol inhibits NF-κB activation and iNOS → lowers NO and TNF-α in macrophages Anti-inflammatory effects in skin and liver (e.g., kahweol reduced UV-induced skin inflammation by curbing COX-2) Bias macrophages toward M2 phenotype via PPARγ activation	[17,28,29,33]

Note: NF-κB = Nuclear Factor kappa B; iNOS = inducible Nitric Oxide Synthase; SOD = Superoxide Dismutase; GSH = Glutathione; PPARγ = Peroxisome Proliferator-Activated Receptor gamma; NOX = NADPH Oxidase; SCFA = Short-Chain Fatty Acid; GPR43 = G-protein-coupled Receptor 43; COX-2 = Cyclooxygenase-2; M2 = Alternatively Activated Macrophage Phenotype.

**Table 2:** Ion channel targets modulated by spent coffee ground bioactives and their relevance in chronic immunometabolic diseases.

Target (Cells/Tissue)	Modulation by SCG Bioactives	Evidence Level	Implications in Chronic Diseases	References
Ca^2+^ Channels (CRAC, VGCC) [T cells, macrophages, β-cells]	Mild attenuation of Ca^2+^ influx: SCG polyphenols reduce ROS and second messengers driving CRAC opening Possible direct VGCC stabilization in β-cells (supporting insulin release without Ca^2+^ overexcitation)	Preclinical (in vitro/animal)	T2D/Obesity: ↓ Ca^2+^-triggered cytokine secretion (less IL-6, TNF-α); protects β-cells from Ca^2+^ overload under glucotoxic stress CVD: Less Ca^2+^-driven eNOS dysfunction → improved NO-mediated vasodilation Neuroinflammation: Dampened microglial Ca^2+^-dependent activation → slowed neurodegeneration	[30,43,55]
TRPV1 [Sensory neurons, pancreatic islet]	Dual modulation: SCG caffeine and phenolics indirectly activate TRPV1 pathways (thermogenesis, acute insulin release) Prolonged exposure leads to TRPV1 desensitization → reduces chronic pain signaling Antioxidative environment prevents ROS-mediated TRPV1 overactivation	Preclinical; epidemiological	Obesity: Activates TRPV1 on brown fat nerves → ↑ energy expenditure; ↑ leptin and insulin sensitivity (TRPV1 mice have worse obesity/diabetes) Chronic Pain/Inflammation: Desensitizing TRPV1 on nociceptors → lower neurogenic inflammation (reduced substance P/CGRP release) T1D: Controlled TRPV1 activity supports β-cell insulin release acutely, but SCG prevent chronic TRPV1-induced islet stress	[62,63,64]
TRPA1 [Sensory neurons, macrophages]	Antioxidant gating: SCG phenolics scavenge reactive aldehydes (acrolein) and ROS (H_2_O_2_) that normally activate TRPA1 → prevents unwarranted opening Possibly slight direct activation by dietary electrophiles (feruloyl compounds) → desensitization SCFAs (acetate) from SCG fermentation activate vagal TRPA1 → modulates satiety and inflammation indirectly ↓ TRPA1 activity under chronic stress	Preclinical (in vitro/animal)	Inflammation/Pain: ↓ TRPA1-mediated neuroinflammation (arthritis, neuropathy) → less pain and tissue damage Insulin Resistance: TRPA1 activation by metabolic stress curtailed → less aberrant sympathetic output; acute beneficial reflexes (GLP-1 and insulin secretion via TRPA1) remain intact Airways: Lower oxidative TRPA1 activation → less asthma/allergic inflammation	[63,65,66,67,68,69]
TRPM2 [Phagocytes, β-cells]	↓ Overactivation: SCG compounds keep ROS below TRPM2 activation threshold → limits Ca^2+^ influx ↓ TRPM2 expression via anti-inflammatory signaling SCFAs (butyrate) → ↓TRPM2 expression in colon macrophages by epigenetic means Polyphenols → ↓ TRPM2 currents in neutrophil cell lines (mechanism: ADP-ribose scavenging or channel gating interference)	Preclinical (in vitro/animal)	Metaflammation: TRPM2 inhibition in macrophages → ↓ IL-1β, IL-8 in metabolic tissues; dampens chronic inflammatory loop in obesity and fatty liver NLRP3 Inflammasome: TRPM2-mediated Ca^2+^ as a trigger is blocked → less IL-1β β-cells: Protection from ROS-induced TRPM2 overactivation → prevents inappropriate insulin secretion and β-cell apoptosis Stroke/Neuro: Microglial TRPM2 kept in check → potentially ↓ infarct size and neuroinflammatory outcomes	[48,49,70,71]
Kv1.3 K^+^ Channel [Effector T cells]	SCG polyphenols (e.g., caffeic acid) likely ↓ Kv1.3 activity → membrane depolarization in T cells Short-circuits driving force for Ca^2+^ entry → selectively attenuates Teff cell activation Analogous to effects of quercetin and resveratrol (reported Kv1.3 blockers in lymphocytes at micromolar concentrations)	Preclinical; inferential from analogous polyphenols	Autoimmune/Chronic Inflammation: Effector memory T cells (Th1/Th17) require Kv1.3; SCG-mediated blockade lowers IFN-γ and IL-17 output; beneficial in metabolic syndrome (adipose T cell inflammation), T1D (islet-infiltrating T cells), rheumatoid arthritis, MS Regulatory balance: Treg and naïve T cells (less Kv1.3-dependent; use KCa3.1) are relatively spared → balance tips toward regulation	[74,75,76,77]
KATP Channel [Macrophages, β-cells]	SCG postbiotics (SCFAs) and phenolics influence cellular metabolism and nucleotide ratios → favor KATP closure in high-energy states Indirectly mimics effects of KATP blockers (e.g., glibenclamide) in macrophages → anti-inflammatory M2 tilt SCG niacin content and polyphenols preserve β-cell metabolic health → maintain proper KATP cycling (glucose-induced closure for insulin release) Diterpenes or SCG lipids may intercalate into macrophage membranes near KATP (Kir6.x/SUR subunits) and alter nucleotide sensitivity (speculative)	Preclinical; inferential	Macrophage Phenotype: Open KATP (energy-stressed state) drives M1 polarization; SCG helps close KATP → mitigates M1 polarization; ↓adipose tissue inflammation and atherosclerotic plaque instability (plaque macrophages with active KATP are highly inflammatory) Insulin Secretion: Healthy KATP cycling → better glucose-stimulated insulin release; SCG protects KATP machinery from oxidative damage Cardiovascular: KATP opening in vascular smooth muscle causes vasodilation; systemic endothelial function improvements via other channels likely dominate to improve blood pressure	[81,82,83,84,85,86,87]

Notes: CRAC = Calcium Release-Activated Calcium Channel; VGCC = Voltage-Gated Calcium Channel; TRPV1/TRPA1/TRPM2 = Transient Receptor Potential Vanilloid 1/Ankyrin 1/Melastatin 2; Kv1.3 = Voltage-Gated Potassium Channel 1.3; KATP = ATP-Sensitive Potassium Channel; Teff = Effector T Ccell; Treg = Regulatory T Cell; M1/M2 = Classically/Alternatively Activated Macrophage; SCFA = Short-Chain Fatty Acid; CGRP = Calcitonin Gene-Related Peptide. Evidence levels: “Preclinical (in vitro/animal)” = cell culture or animal model data; “Inferential” = mechanistically plausible based on analogous compounds or indirect evidence; “Epidemiological” = human observational data. ↓ denotes downregulation; ↑ = increase/enhancement; → = leads to/results in.

**Table 3:** GPCR targets modulated by spent coffee ground bioactives and their relevance in chronic immunometabolic diseases.

Target [Cell/Tissue]	Modulation by SCG Bioactives	Evidence Level	Implications in Chronic Diseases	References
GPR120 (FFA4) [Macrophages, adipocytes, intestinal L-cells]	Activation by SCG-derived unsaturated fatty acids (linoleic acid ~43–50% of SCG oil) → acts as endogenous GPR120 agonist similarly to fish oil fatty acids Engages GPR120 on tissue macrophages → M2 polarization, IL-10 secretion, TNF-α suppression Cafestol may synergize with omega-3 in macrophages to enhance anti-inflammatory gene expression (possible GPR120 crosstalk) GPR120 can signal via β-arrestin to inhibit NF-κB; coffee phenolics may favor β-arrestin route over Gq	Preclinical (in vitro/animal)	Obesity/T2D: GPR120 activation in adipose tissue → reduces crown-like structure inflammation; improves insulin sensitivity (increased GLUT4 translocation and adiponectin); prevents adipocyte hypertrophy Glycemic control: GPR120 on intestinal L-cells → stimulates GLP-1 release CVD: GPR120 on plaque macrophages → inhibits MMP secretion and pro-inflammatory cytokines → potential plaque stabilization	[12,63,88]
GPR43 (FFA2) [Colonic epithelium, gut macrophages, neutrophils, adipocytes, β-cells]	Activated by SCFAs (acetate, propionate, butyrate) generated from SCG melanoidin and fiber fermentation SCG acts as a prebiotic: elevates luminal SCFAs → engages GPR43 on gut macrophages → induces IL-18 and mucosal-healing cytokines; reinforces gut barrier and reduces systemic endotoxemia GPR43 on neutrophils → more controlled recruitment (GPR43 KO mice show exacerbated inflammation in colitis and arthritis) GPR43 on β-cells → enhances insulin secretion in response to FFAs in high-fat-diet conditions GPR43 activation in adipocytes → prevents hypertrophy and insulin resistance (directs energy to muscles by reducing insulin signaling in adipose)	Preclinical; human (dietary fiber studies)	Gut/Immune: Reinforces gut barrier; ↓ systemic endotoxemia; ↑colonic regulatory T cell responses T2D/Obesity: ↑ glucose homeostasis and insulin dynamics; reduces adipose inflammatory tone (lower liver and adipose IL-6, CRP) CVD: GPR43 activation reduces adhesion molecule expression on endothelium → ↓ monocyte recruitment to plaques; GPR43 KO mice show worse arterial inflammation on high-fat diet	[21,89,90,91,92]
Adenosine A_2_A Receptor [T cells, dendritic cells, macrophages, microglia, vascular endothelium]	Fine-tuning via caffeine antagonism: blocks A_2_A → temporarily heightens immune surveillance; chronic use upregulates adenosine receptors → net anti-inflammatory effect when caffeine absent SCG contains small amounts of adenosine/nucleosides (especially espresso SCG) → may directly activate A_2_A Melanoidins may bind adenosine or influence its metabolism SCG diterpenes (cafestol) have adenosine-like effects in some contexts (via PPAR crosstalk with A_1_ receptor signaling) SCG phenolics may act as partial A_2_A agonists → immunotolerant state without full cAMP spike A_2_B receptors (pro-inflammatory in high glucose) may be antagonized by caffeine	Preclinical; epidemiological	Neuroinflammation/PD/AD: A_2_A blockade on microglia prevents overactivation → ↓ neurotoxic cytokines; associated with lower risk of Parkinson’s and Alzheimer’s disease; preserves microglial debris-clearing function Macrophage programming: A_2_A stimulation ↑ cAMP → ↓ TNF-α, IL-12; ↑ IL-10; ↓ pro-inflammatory microglial activation CVD/Diabetes: A_2_B antagonism by caffeine → ↑ endothelial outcomes in high-glucose conditions Immune balance: Fine-tuning avoids both immunosuppression and chronic overactivation	[26,93,94,95,96,97]

Notes: GPCR = G-protein-coupled Receptor; FFA2/FFA4 = Free Fatty Acid Receptor 2/4 (GPR43/GPR120); SCFA = Short-Chain Fatty Acid; GLP-1 = Glucagon-Like Peptide-1; MMP = Matrix Metalloproteinase; M1/M2 = Classically/Alternatively Activated Macrophage; KO = Knockout; cAMP = cyclic Adenosine Monophosphate; PPAR = Peroxisome Proliferator-Activated Receptor; PD = Parkinson’s Disease; AD = Alzheimer’s Disease. Evidence levels: “Preclinical (in vitro/animal)” = cell culture or animal model data; “Human (dietary fiber studies)” = clinical intervention data for SCFAs/fiber broadly; “Epidemiological” = human observational data. ↓ denotes downregulation; ↑ = increase/enhancement; → = leads to/results in.

## Data Availability

No new data were created or analyzed in this study. Data sharing is not applicable to this article.

## Abbreviations

Abbreviation	Full Term
SCG	Spent coffee grounds
GPCR	G-protein-coupled receptor
ROS	Reactive oxygen species
TRP	Transient receptor potential (channel)
CGA	Chlorogenic acid
CRAC	Calcium release-activated calcium (channel)
SCFA	Short-chain fatty acid
T2D	Type 2 diabetes
CVD	Cardiovascular disease
NF-κB	Nuclear factor kappa B
NLRP3	NOD-, LRR- and pyrin domain-containing protein 3
GPR43/GPR120	G-protein-coupled receptor 43/120 (also FFAR2/FFAR4)
TRPV1/TRPA1/TRPM2	TRP vanilloid 1/ankyrin 1/melastatin 2
Kv1.3	Voltage-gated potassium channel 1.3
KATP	ATP-sensitive potassium channel
SOCE	Store-operated calcium entry
UAE/MAE/SFE/EAE	Ultrasound-/microwave-/supercritical fluid-/enzyme-assisted extraction
DPPH/FRAP	2,2-diphenyl-1-picrylhydrazyl/Ferric reducing antioxidant power (antioxidant assays)
EFSA	European Food Safety Authority
GRAS	Generally Recognized As Safe
